# Anion Component Engineering of Spontaneous Perovskite Passivators for Energy Alignment Modulations in Perovskite Solar Cells

**DOI:** 10.1002/smll.202512937

**Published:** 2026-01-25

**Authors:** Naoyuki Nishimura, Hiroaki Tachibana, Takurou N. Murakami

**Affiliations:** ^1^ National Institute of Advanced Industrial Science and Technology (AIST) Tsukuba Ibaraki Japan

**Keywords:** bis(fluorosulfonyl)amide (FSA), bis(pentafluoroethylsulfonyl)amide (PFSA), bis(trifluoromethylsulfonyl)amide (TFSA), photovoltaics, spontaneous heterointerface modulator (SHM)

## Abstract

Emerging alkyl‐primary‐ammonium‐bis(trifluoromethanesulfonyl)imides (RA‐TFSIs), used as additives for hole‐transport materials (HTMs) in perovskite solar cells (PSCs), exhibit prominent functions as spontaneous perovskite passivators; RA cations spontaneously suppress defects over the perovskite surface during HTM deposition, while TFSI anions, remaining in the HTM bulk, enhance the hole mobility of the HTMs. In RA‐TFSIs, their cation components have been developed, whereas the anion components remain unexplored. The most commonly used TFSI is a series of bis(fluorosulfonyl)imides (e.g., bis(fluorosulfonyl)imide (FSI) and bis(pentafluoroethanesulfonyl)imide (PFSI)), yet the effectiveness of bis(fluorosulfonyl)imides remains controversial. In this study, the functions of *n*‐octylammonium (OA)‐bis(fluorosulfonyl)imides (i.e., OA‐FSI, OA‐TFSI, and OA‐PFSI) are verified. The anion components manipulate ionization energies (IEs) of the HTM, thereby effectively modulating the energy alignment in PSCs. The larger C‐F moieties in bis(fluorosulfonyl)imides, from FSI to TFSI and PFSI, led to deeper ionization energies (IEs), presumably owing to their spatially more expanded electron delocalization. In particular, the FSI‐based additives led to shallow IEs of the HTMs and exhibited some benefits for the photovoltaic performance of PSCs, including their stability, which have rarely been discussed. This work provides novel insights into HTM additives, which may be a bottleneck of n‐i‐p structured PSCs, leading to further advancements in PSCs.

## Graphical abstract

*n*‐Octylammonium bis(fluorosulfonyl)imides (OA‐FSI, OA‐TFSI, and OA‐PFSI), used as additives for spiro‐OMeTAD hole‐transport materials (HTMs) in perovskite solar cells (PSCs), allow spontaneous perovskite passivation during HTM deposition. Anion component engineering of the OA‐bis(fluorosulfonyl)imides effectively modulate energy alignment in PSCs via manipulation of the interactions between spiro‐OMeTAD and bis(fluorosulfonyl)imides.

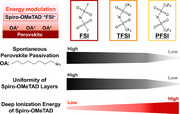

## 1 Introduction

Additives for hole‐transport materials (HTMs) in perovskite solar cells (PSCs) play an important role in ensuring efficient PSCs with n‐i‐p structures [1, 2, 3, 4]. Because the organic semiconductors used as HTMs in PSCs possess insufficient hole mobilities, additives that enhance the hole mobilities are essential. For spiro‐OMeTAD HTM with lithium bis(trifluoromethanesulfonyl)imides (Li‐TFSI), which are the major HTM and additive, the enhancement of the hole mobility of the HTM occurs as described below: spiro‐OMeTAD cationic radicals (spiro‐OMeTAD^·+^), which increase the hole mobility of the spiro‐OMeTAD layers, are formed via oxidation Equation (1), and the cationic radicals are stabilized with the TFSI anion Equation (2) [3, 4, 5, 6, 7, 8].

(1) $$2\mathrm{Spiro}-\mathrm{OMeTAD}+1/2O_{2}\to 2\mathrm{Spiro}-\mathrm{OMeTA}D^{+}+O^{2^{-}}$$

(2) $$\mathrm{Spiro}-\mathrm{OMeTA}D^{+}+\mathrm{TFS}I^{-}\to \mathrm{Spiro}-\mathrm{OMeTA}D^{•+}\mathrm{TFS}I^{-}$$

**FIGURE 1:**
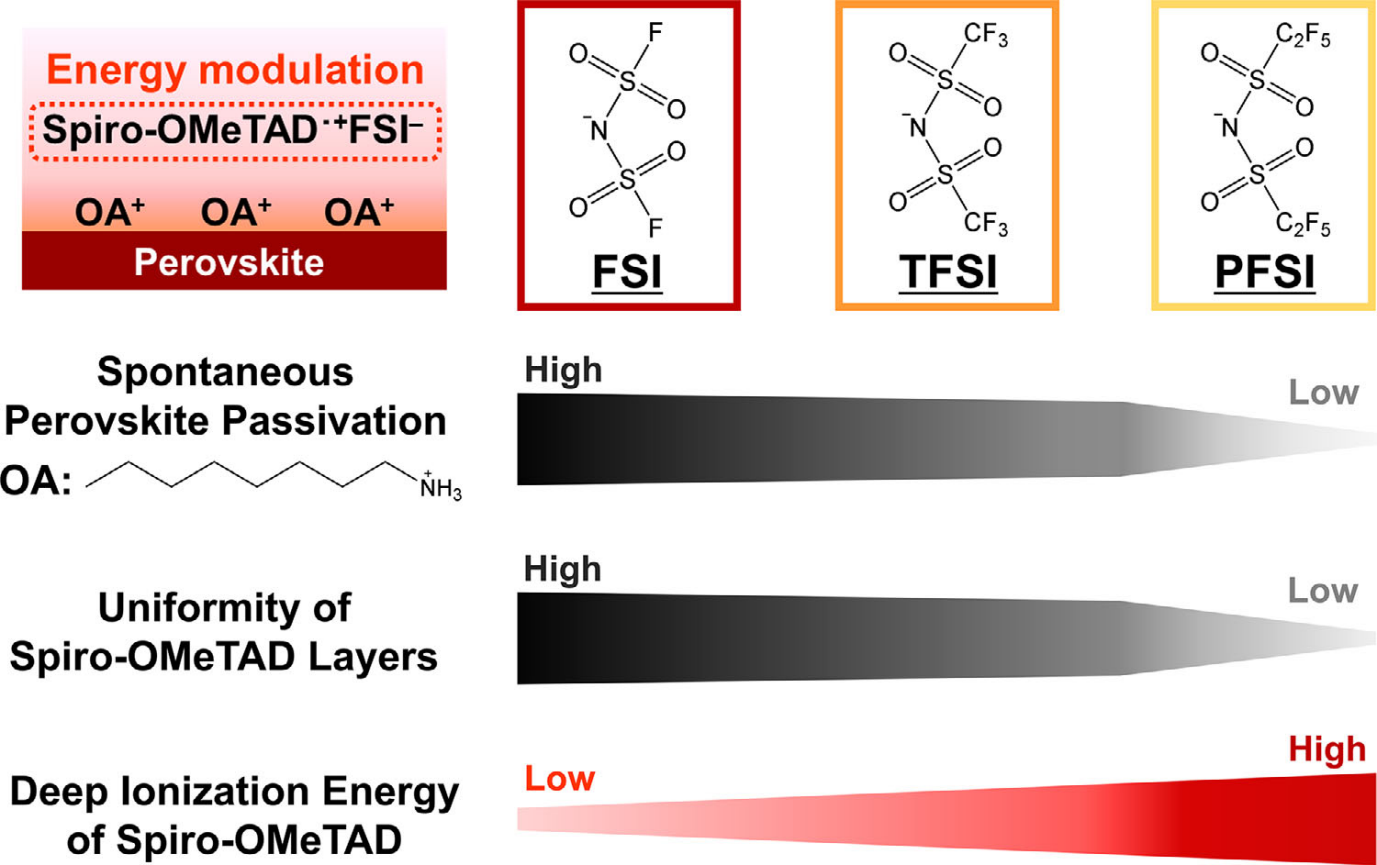
Schematic of anion component engineering for OA‐based spontaneous perovskite passivators.

Thus, HTM additives consisting of TFSI anions enhance the hole mobility of HTM. Although TFSI is the anion used most commonly in HTM additives, the functions enabled by TFSI anions are limited. Therefore, engineering the anion component in HTM additives is key to the further development of PSCs. TFSI is categorized into the bis(fluorosulfonyl) imide group, which includes bis(fluorosulfonyl)imide (FSI) and bis(pentafluoroethanesulfonyl)imide (PFSI) (Figure 1). The use of a series of bis bis(fluorosulfonyl)imides as HTM additives is of interest and will lead to further advances in PSCs [9, 10, 11]. However, the effects of different FSI anions on HTM doping and PSC performance have remained debatable; for Li‐based bis(fluorosulfonyl)imides, some reports have indicated that FSI and/or PFSI are superior to TFSI [9, 10], while others insist that TFSI is optimal among bis(fluorosulfonyl)imides [11]. Therefore, further clarification of these effects is required.

One reason for this discrepancy could be the use of Li‐based materials. Li species affect the photovoltaic (PV) performance of PSCs and are known to be detrimental [12, 13, 14, 15, 16, 17, 18, 19, 20, 21, 22]. The mixed effects of Li and modulated anions could complicate differentiating their contributions to the PV performance. Therefore, investigating the effects of bis(fluorosulfonyl)imides with Li‐free HTM additives is a promising strategy. Recently, alkyl‐primary‐ammonium‐based TFSIs (RA‐TFSIs) [8, 23, 24, 25, 26, 27, 28, 29, 30, 31, 32, 33] have emerged as Li‐free HTM additives, and archetypal *n*‐octylammonium TFSI (OA‐TFSI) [23, 24, 25, 26, 27] has demonstrated its usefulness. In addition to its Li‐free nature, RA‐TFSI is advantageous for enhancing PV performance of PSCs via their spontaneous perovskite passivation function; during deposition of HTM solution containing RA‐TFSIs, the RA cations spontaneously suppress the defects over the perovskite surface, exploiting the large adsorption energies of the RA cations on perovskite surfaces (e.g., 1.88 eV for OA cations [34]), while TFSI anions remain in the HTM bulk, enhancing its hole mobility (Figure S1). Thus, using the RA‐TFSI spontaneous perovskite passivators, additional passivation processes that conventional RA halide passivators (e.g., RAIs) require [34, 35, 36, 37, 38, 39, 40, 41, 42, 43, 44, 45, 46, 47, 48, 49, 50, 51, 52, 53] are omitted; RA‐TFSI is beneficial in terms of enhancing the process efficiency of PSC fabrication, similar to other spontaneous heterointerface modulators [8, 54, 55, 56, 57, 58, 59, 60, 61, 62, 63]. Despite their promising functions, however, RA‐TFSIs are nascent, and their components have yet to be explored. In particular, although the effects of cationic components have been investigated, their anionic components have not yet been explored.

In this study, we engineered anionic components in OA‐based spontaneous perovskite passivators to further understand the functions of bis(fluorosulfonyl)imides and the development of spontaneous perovskite passivators (Figure 1). OA‐FSI and OA‐PFSI were newly synthesized, and their effects as HTM additives were compared with those of OA‐TFSI. As OA‐TFSI has been the most investigated RA‐TFSI thus far [8, 23, 24, 25, 26, 27], OA was chosen as the cation component. For reference, the effects of Li‐FSI, Li‐TFSI, and Li‐PFSI were also investigated in our system. As a consequence, engineering of the anion components allowed substantial modulation of the energy alignment in PSC, which affected their PV performance beyond the effects of the most commonly used TFSI‐based additives, providing clues for the further development of PSCs.

## 2 Results and Discussion

Novel OA‐FSI and OA‐PFSI HTM additives were successfully synthesized via an ion‐exchange method [23] and were in the liquid state at room‐temperature. The OA‐TFSI from a previously reported batch [23] was used in this study. Thus, the series of OA‐bis(fluorosulfonyl)imides (OA‐FSI, OA‐TFSI, and OA‐PFSI; denoted OA‐based additives afterward) was prepared. The specific nature of the TFSI anion is that of electron delocalization, which contributes to efficient stabilization of the spiro‐OMeTAD cationic radicals. In terms of anion modulations, with an increase in the size of C‐F moieties in bis(fluorosulfonyl)imides (i.e., from FSI to TFSI, PFSI), the delocalized electrons expand more in the larger physical spaces [9, 10, 11]. The spatial expansion of delocalized electrons from FSI to TFSI and PFSI has been elucidated by theoretical studies and corroborated by experimental results [64, 65, 66, 67, 68, 69]. For instance, expansion of delocalized electrons in the TFSI anion relative to FSI was substantiated by experimental identification of ion coordination using solution‐state NMR and Raman spectroscopy with theoretical studies [68]. Moreover, among FSI, TFSI, and PFSI, larger nanodomains associated with longer fluorous moieties in bis(fluorosulfonyl)imides were confirmed by high‐energy X‐ray scattering [66]. The magnitude of the spatial expansion in delocalized electron delocalization in bis(fluorosulfonyl)imides affects their function as HTM additives, which may involve spontaneous perovskite passivation.

**FIGURE 2:**
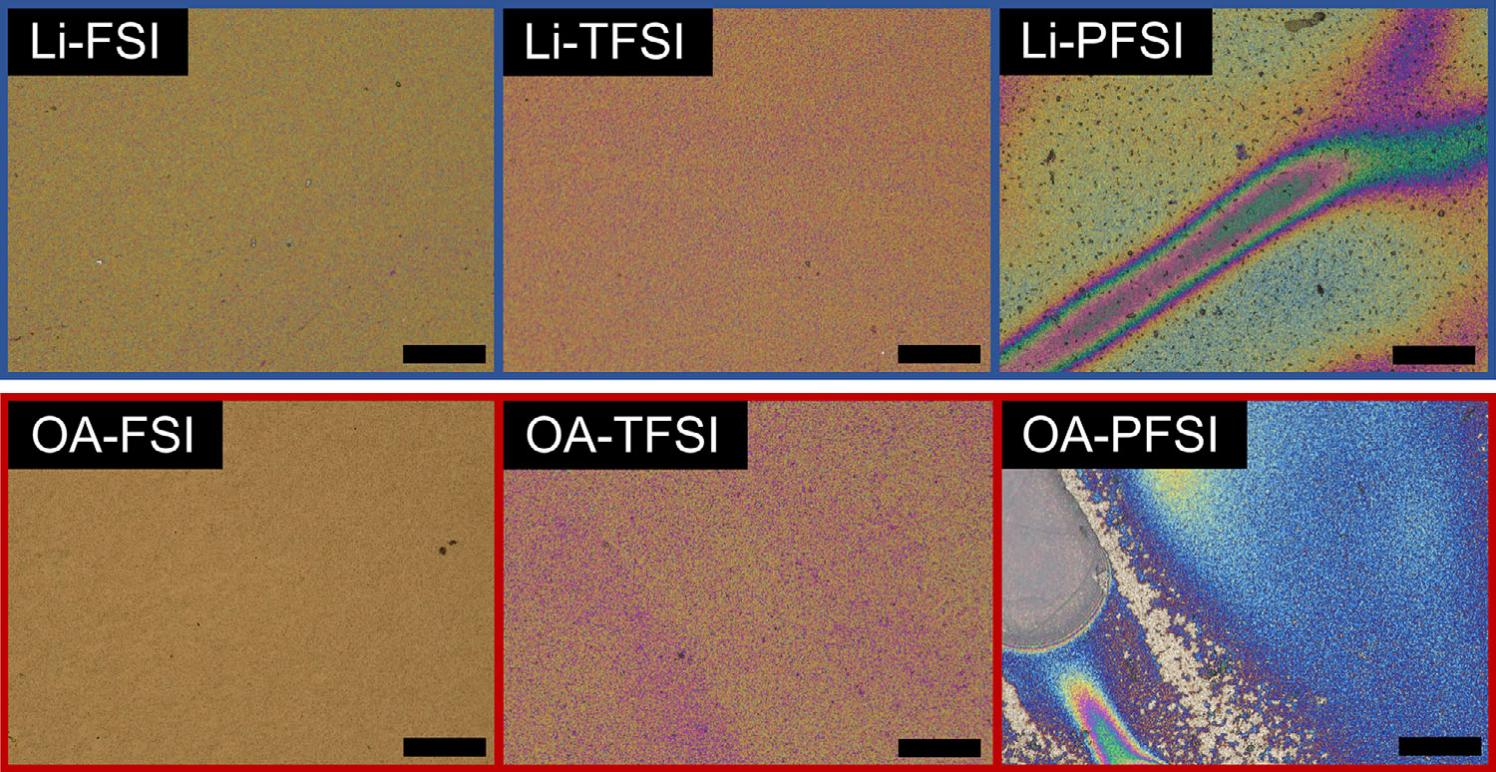
Microscopic views of HTM layers with Li‐ and OA‐based bis(fluorosulfonyl)imide additives; the scale bars denote 50 µm (Magnified pictures: Figure S2).

Figure 2 shows microscopic images of HTMs comprising Li‐ and OA‐based bis(fluorosulfonyl)imide additives. The HTM layers comprising FSI‐ and TFSI‐based additives have almost uniform morphologies, whereas those with PFSI‐based additives exhibit color gradations and a number of voids. This trend was also observed in the magnified images (Figure S2). Therefore, the increase in the size of C‐F moieties in bis(fluorosulfonyl)imides influenced the morphologies of the HTM layers, and the PFSI‐based additives caused a deterioration of the morphologies of the HTM layers. This trend is consistent with that of previous reports; HTM layers with Li‐PFSI additives resulted in ununiform morphologies due to the aggregation of Li‐PFSI induced by enhanced hydrophobicity and lipophobicity of PFSI [10, 11]. While changes in HTM morphologies affect the PV performance of PSCs, the anionic components in the HTM additives modulate other properties, especially the energy alignments in PSCs, providing profound insights into HTM additives.

**FIGURE 3:**
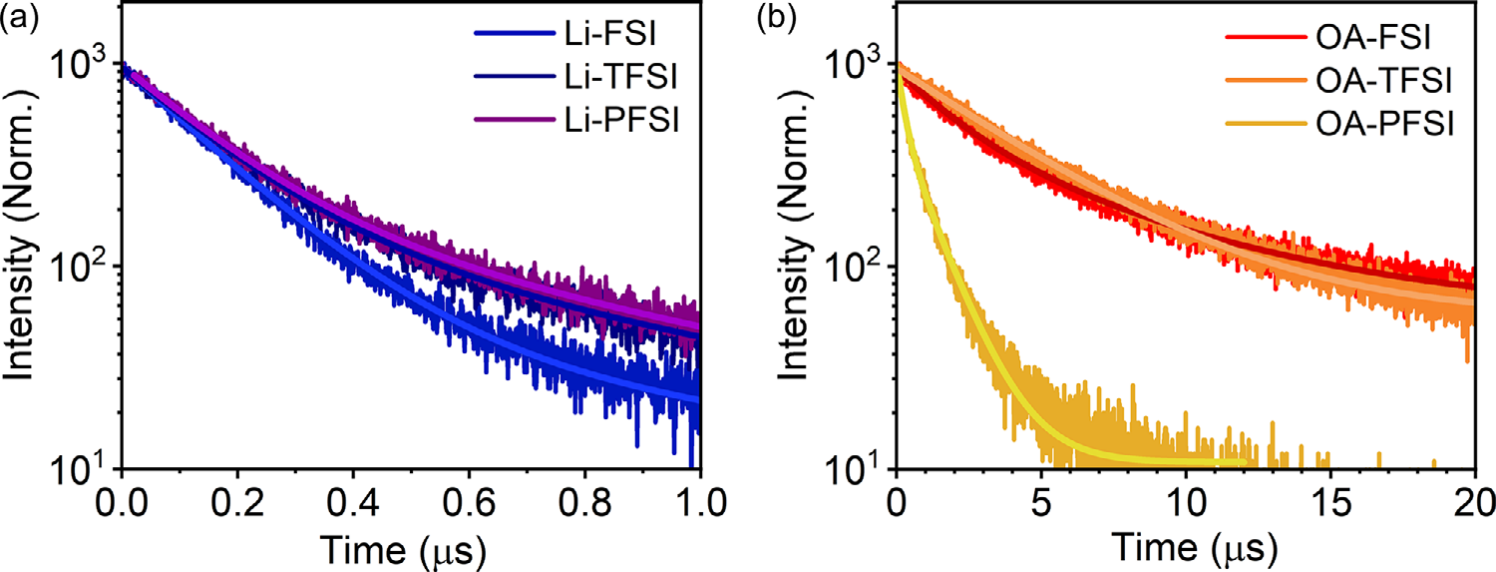
PL decays of perovskite monolayer samples after removal of HTMs with (a) Li‐ and (b) OA‐based additives.

For OA‐based additives, spontaneous perovskite passivation is their key function. Thus, characteristics regarding perovskite passivation by OA cations are investigated in terms of surface wettability and carrier dynamics. Figure S3 illustrates the contact angles (CAs) of water droplets to perovskite layers after removal of HTM comprising the Li‐ and OA‐based additive. Although the samples comprising Li‐based additives had a CA of 46°–51°, the OA‐based additives markedly increased the CAs to 72°–82°, indicating that OA cations successfully covered perovskite surfaces via spontaneous perovskite passivation processes during HTM depositions [8, 23, 25, 26, 29, 30, 31]. Figure 3 depicts the photoluminescence (PL) lifetimes of the perovskite layers after removal of HTM layers [23, 26, 27, 28, 29, 30, 31] comprising the additives. A longer PL lifetime strongly suggests less defects, acting as carrier traps, over the perovskite surfaces [55, 70]. All samples containing Li‐based additives exhibited short PL lifetimes up to 265 ± 5 ns. By contrast, the OA‐based HTM additives significantly increased PL lifetimes. Specifically, the OA‐FSI and OA‐TFSI exhibited substantially longer PL lifetimes of 4.19 ± 0.18 µs and 4.42 ± 0.01 µs, respectively, compared with those of the corresponding Li‐based additives (Li‐FSI: 0.247 ± 0.006 µs and Li‐TFSI: 0.257 ± 0.010 µs), respectively, indicating that spontaneous perovskite passivation effectively suppressed the defects over the perovskite surfaces. Although the OA‐PFSI HTM additive also increased the PL lifetime from 265 ± 5 ns to 695 ± 6 ns, the increase was not significant, relative to that of the other OA‐based additives. This is perhaps because the cationic nature of OA cations, which provides a large adsorption energy of OA on perovskite surfaces, is key for efficient spontaneous perovskite passivation, whereas the electrons delocalized in the relatively large physical space of the PFSI anions [9, 10, 11] could frequently interact with OA cations during the HTM deposition process, decreasing their perovskite passivation functions. Indeed, previous works elucidated that the coexistence of anions and/or losing the cationic nature of OA significantly decreased the spontaneous perovskite passivation function of OA cations [23, 25]. Overall, spontaneous perovskite passivation effects caused by OA cations in OA‐based additives were observed, but the effect of OA‐PFSI was less than that of other OA‐based additives, perhaps due to the spatial expansion of the delocalized electron in the PFSI anion.

The additives contribute to stabilization of spiro‐OMeTAD cationic radicals (Equation (2), and a higher concentration of spiro‐OMeTAD cationic radicals likely leads to deeper ionization energies (IEs) of HTMs [3]. Thus, engineering of additives can modulate the IEs of HTMs. Manipulating the IE of HTM is pivotal for practical PSCs because it determines the energy gap between perovskite photoabsorbers and HTMs. The valence band (V.B.) top of perovskites is required to be deeper than the IE of HTMs for efficient hole transfer from perovskites to HTMs with sufficient driving energy for the hole transfer. Conversely, an excessively large energy gap between perovskite photoabsorbers and HTMs will result in energy loss via hole transfer. Typically, using the conventional Li‐TFSI, the IE range of spiro‐OMeTAD HTM is prone to being limited and to providing sufficient driving energy for hole transfer. Thus, the prevailing opinion might be that the deeper IEs of HTMs are beneficial for obtaining highly efficient PSCs with low energy losses during hole transfer. However, engineering the anion component in HTM additives enables notable changes in the IEs and exceeds the limitation of TFSI additives; this phenomenon will be discussed in the following sections, highlighting the importance of energy alignment modulation in PSCs.

**FIGURE 4:**
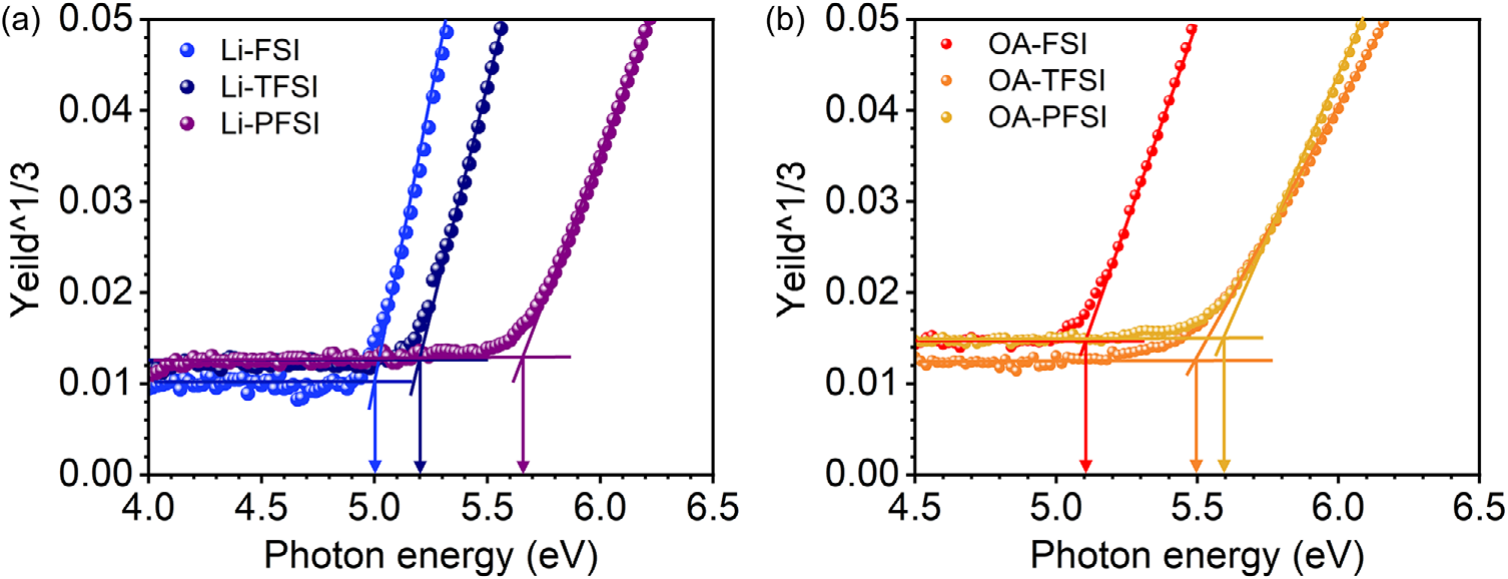
PYS profiles of HTM layers with (a) Li‐ and (b) OA‐based additives.

Figure 4 displays the results of photon yield spectroscopy (PYS) measurements, illustrating the IEs of the materials. The HTM additives changed the IEs of the HTMs, and their anion components significantly influenced the IEs. Among the Li‐based additives, Li‐FSI exhibited a shallower IE (5.00 eV compared with that of Li‐TFSI (5.21 eV)), whereas Li‐PFSI exhibited the deepest IE of 5.66 eV. Thus, with an increase in the size of C‐F moieties in bis(fluorosulfonyl)imides (i.e., from FSI to TFSI and PFSI) of the HTM additives, the IEs of the resulting HTMs increased. This trend is attributable to electron delocalization in bis(fluorosulfonyl)imides [9, 10, 11]; with a larger size of C‐F moieties in bis(fluorosulfonyl)imides, electrons are more delocalized and thereby more effectively stabilize the cationic radicals of spiro‐OMeTAD, resulting in deeper IEs.

**FIGURE 5:**
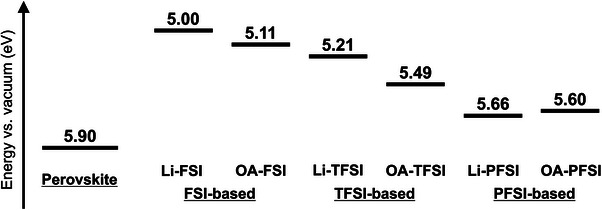
Energy alignments of PSC components using Li‐ and OA‐based HTM additives.

The OA‐based additives tended to display deeper IEs than that of the corresponding Li‐based additives, while the trends in terms of the changing anion components were similar. OA‐FSI had the shallowest IE of 5.11 eV, while OA‐TFSI and OA‐PFSI exhibited IEs of 5.49 and 5.60 eV, respectively. Therefore, the IE trends of the OA‐based additives were determined based on their component anions. The substantially deeper IEs of the HTMs comprising OA‐FSI and OA‐TFSI additives than that of those with the corresponding Li‐based additives may be attributed to the absence of counter cations in the additives [8, 23, 25]. When Li‐based additives are used, the presence of positively charged counter cations (i.e., Li^+^ cations) can hamper the formation of HTM cationic radicals via oxidation reactions with electron acceptors (e.g., O_2_ molecules in Equation (1)) and interfere with the stabilization of HTM cationic radicals by TFSI Equation (2). However, OA‐based additives mitigate this issue because of the absence of counter cations in the HTM bulk, leading to deeper IEs, as observed in OA‐FSI and OA‐TFSI. The IE value of OA‐PFSI did not exceed that of Li‐PFSI, suggesting that the absence of counter cations in the HTM bulk did not affect the PFSI anions. This is presumably because PFSI anions suffice to stabilize spiro‐OMeTAD cationic radicals even in the presence of the counter cations, and therefore, the obstructions by the counter cations became negligible. The summary of IEs is depicted in Figure 5 (Figure S4: the PYS profile of the pristine perovskite layer). Overall, compared with that of conventional TFSI‐based additives, FSI‐based additives exhibited considerably shallower IEs of HTMs, whereas PFSI‐based additives caused deeper IEs. In particular, the shallow IEs resulting from the FSI additives exhibited some advantages in the PV performance involving long‐term stability, which is rare, and might contradict the prevailing school of thought, as will be discussed later.

**FIGURE 6:**
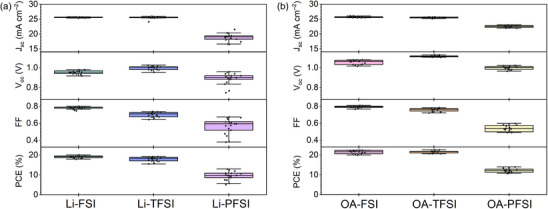
PV parameter distributions of PSCs with Li‐ and OA‐based bis(fluorosulfonyl)imides in backward scan (Figure S5: forward scan).

**FIGURE 7:**
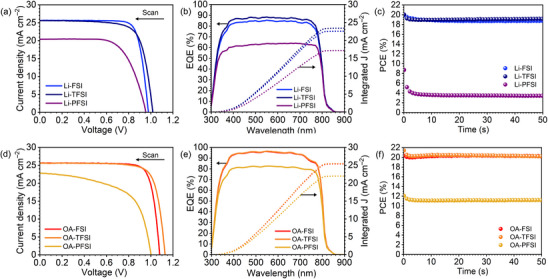
PV performance of PSCs with Li‐ and OA‐ based additives. *J*–*V* curves in backward scan (forward scan: Figure S6) for PSCs with (a) Li‐based (d) OA‐based HTM additives. EQE spectra for PSCs with (b) Li‐based (e) OA‐based HTM additives. QSS‐PCEs (c) at 0.83, 0.85, 0.73 V for Li‐FSI, Li‐TFSI and Li‐PFSI, respectively; (f) at 0.92, 0.94, 0.74 V for OA‐FSI, OA‐TFSI and OA‐PFSI, respectively.

Figures 6 and 7 display the PV performances of the PSCs comprising Li‐ and OA‐based bis(fluorosulfonyl)imide additives. Overall, the PSCs containing OA‐based bis(fluorosulfonyl)imide additives exhibited a higher PV performance relative to those comprising the corresponding Li‐based additives, owing to an increase in open‐circuit voltage (*V*_oc_), spontaneous perovskite passivation by OA cations; however, irrespective of the dopant cation (i.e., Li^+^ or OA^+^), PSCs comprising the bis(fluorosulfonyl)imide series exhibited similar anion‐dependent trends.

Among Li‐based additives, PSCs comprising Li‐FSI and Li‐TFSI exhibited similar PCE values of 19.1 ± 0.6% and 17.9 ± 1.1%, respectively, in the backward scan (best performing: 20.1% and 19.3%), respectively. Similarly, the PSCs comprising Li‐FSI and Li‐TFSI exhibited QSS‐PCE of 18.7% and 19.2%, respectively. Nevertheless, the PV parameter trends differed, presumably because of the difference in the energy alignments modulated by the dopant anions (i.e., FSI and TFSI). PSCs comprising Li‐TFSI exhibited a higher *V*_oc_ of 1.00 ± 0.02 V (best performing: 1.02 V) in a backward scan than those comprising Li‐FSI (0.96 ± 0.02 V; best performing: 0.98 V) owing to the deeper IE of HTM, which reduced energy loss via hole transfer from the perovskite to the HTM. Conversely, PSCs comprising Li‐FSI exhibited higher fill factor (FF) values of 0.78 ± 0.01 (best performing: 0.80) than those comprising Li‐TFSI (0.70 ± 0.03; best performing: 0.74), which can be attributed to the larger driving energy required for hole transfer from the perovskite to the HTM. Thus, energy alignment modulation by the dopant anions (Figures 4 and 5) can be compensated for; a larger energy gap between the perovskites and HTMs is advantageous for obtaining higher FF values, whereas a smaller energy gap is beneficial for larger *V*_oc_ values. The integrated current density (*J*) calculated from the external quantum efficiency (EQE) spectra (Figure 7b) was significantly different from the short‐circuit current density (*J*_sc_) obtained from the current density–voltage (*J*–*V*) curves. Specifically, the integrated *J* for the PSCs comprising Li‐FSI and Li‐TFSI were calculated as 22.5 mA cm^−2^ and 23.4 mA cm^−2^, respectively, which were large (25.6 mA cm^−2^ and 25.7 mA cm^−2^) with a difference over 9%. This difference is presumably due to the presence of numerous carrier traps in PSCs comprising Li‐based additives, and the weak excitation conditions in the EQE measurements (< 1 mW cm^−2^) [30, 70]. The small number of generated carriers under the conditions used for EQE measurement was mostly trapped in the carrier traps in the PSCs comprising Li‐based additives, decreasing the EQE values, while the number of generated carriers under one sun irradiation, used during the *J*–*V* curve measurement, filled the carrier traps and negligibly affected the *J*_sc_ values. However, spontaneous perovskite passivation using OA‐based additives addressed this issue and the *J* calculated from the EQE spectra and *J*_sc_ in the *J*–*V* curves were similar, as will be shown later.

Furthermore, PSCs comprising Li‐PFSI exhibited a substantially lower PCE of 9.5 ± 2.1% in backward scan (best performing: 13.0%, QSS‐PCE: 3.4%) than those of PSCs comprising Li‐FSI and Li‐TFSI, which exhibited lower values of *J*_sc_, *V*_oc_, and FF presumably owing to the non‐uniform HTM layers with Li‐PFSI [10, 11]. The considerably lower *J*_sc_ and FF values were attributed to the very low driving energy required for hole transfer from the perovskite to the HTMs (Figures 4 and 5). The observed PV performance trends for Li‐FSI, Li‐TFSI, and Li‐PFSI are in line with a previous report [11].

The OA‐series dopants exhibited similar trends with respect to the dopant anions. PSCs comprising either OA‐FSI or OA‐TFSI clearly showed increased PV performance compared with those with the corresponding Li‐based dopants owing to spontaneous perovskite passivation, which enhanced *V*_oc_. For instance, OA‐FSI increased *V*_oc_ to 1.06 ± 0.02 V (best performing: 1.08 V), from Li‐FSI with a *V*_oc_ of 0.96 ± 0.02 V (best performing: 0.98 V), while OA‐TFSI increased *V*_oc_ to 1.12 ± 0.01 V (best performing: 1.13 V) compared with that of Li‐TFSI of 1.00 ± 0.02 V (best performing: 1.02 V). Even with the spontaneous perovskite passivation effects with the OA components in the dopants, PSCs comprising OA‐FSI and OA‐TFSI exhibited similar PCE values of 21.5 ± 0.8% and 21.6 ± 0.6% in the backward scan (best performing: 22.4% and 22.6%), respectively. Although the PSCs comprising OA‐FSI exhibited larger hysteresis in the *J*–*V* curves (Table 1), the QSS‐PCEs of the PSCs comprising OA‐FSI and OA‐TFSI showed similar values (both 20.2%). Moreover, the trends in the PV parameters of the PSCs comprising OA‐FSI and OA‐TFSI were similar to those of the PSCs comprising Li‐FSI and Li‐TFSI. The PSCs comprising OA‐TFSI displayed higher *V*_oc_ values of 1.12 ± 0.01 V (best performing: 1.13 V) than those comprising OA‐FSI with a *V*_oc_ of 1.06 ± 0.02 V (best performing: 1.08 V). Conversely, PSCs comprising OA‐FSI exhibited higher FF values of 0.79 ± 0.01 (best performing: 0.81) than those comprising OA‐TFSI with an FF of 0.76 ± 0.02 (best performing: 0.78). This trend is attributable to the energy alignment modulated by the HTM additives, highlighting its impact on PV performance. Notably, the integrated *J* calculated from the EQE spectra (Figure 7e) was consistent with the *J*_sc_ obtained from the *J*–*V* curves; the integrated *J* for the PSC comprising OA‐FSI and OA‐TFSI were calculated as 25.4 mA cm^−2^ and 25.5 mA cm^−2^, respectively, closely matching the *J*_sc_ from the *J*–*V* curves (both 25.6 mA cm^−2^) with a difference of 1%. Although the Li‐based additives exhibited a relatively large difference between the integrated *J* in the EQE and *J*_sc_ in the *J*–*V* curves, presumably due to the presence of carrier traps, the well‐matched integrated *J* and *J*_sc_ in the *J*–*V* curves for the OA‐based additives support effective perovskite passivation by OA cations, eliminating carrier traps.

**TABLE 1:** PV parameters of PSCs with Li‐ and OA‐based HTM additives

Additive	Scan	Average / Best	*J*_sc_ (mA/cm^2^)	*V*_oc_ (V)	FF	PCE (%)
Li‐FSI	Backward	Best	25.7	0.98	0.80	20.1
		Average	25.6 ± 0.1	0.96 ± 0.02	0.78 ± 0.01	19.1 ± 0.6
	Forward	Best	25.7	0.81	0.57	11.9
		Average	25.6 ± 0.1	0.76 ± 0.03	0.56 ± 0.04	10.9 ± 1
Li‐TFSI	Backward	Best	25.6	1.02	0.74	19.3
		Average	25.5 ± 0.4	1.00 ± 0.02	0.70 ± 0.03	17.9 ± 1.1
	Forward	Best	25.7	0.94	0.61	14.6
		Average	25.6 ± 0.4	0.89 ± 0.03	0.55 ± 0.03	12.5 ± 1
Li‐PFSI	Backward	Best	20.4	0.96	0.67	13.0
		Average	18.9 ± 1.2	0.89 ± 0.06	0.56 ± 0.09	9.5 ± 2.1
	Forward	Best	16.6	0.68	0.19	2.1
		Average	17.4 ± 1.9	0.67 ± 0.11	0.2 ± 0.02	2.4 ± 0.6
OA‐FSI	Backward	Best	25.6	1.08	0.81	22.4
		Average	25.7 ± 0.2	1.06 ± 0.02	0.79 ± 0.01	21.5 ± 0.8
	Forward	Best	25.7	1.02	0.69	17.9
		Average	25.7 ± 0.2	0.98 ± 0.03	0.66 ± 0.02	16.6 ± 1.0
OA‐TFSI	Backward	Best	25.6	1.13	0.78	22.6
		Average	25.5 ± 0.2	1.12 ± 0.01	0.76 ± 0.02	21.6 ± 0.6
	Forward	Best	25.7	1.08	0.67	18.6
		Average	25.6 ± 0.2	1.07 ± 0.01	0.66 ± 0.02	18.1 ± 0.5
OA‐PFSI	Backward	Best	22.8	1.00	0.59	13.5
		Average	22.5 ± 0.3	1.00 ± 0.02	0.54 ± 0.04	12.1 ± 1.0
	Forward	Best	23.1	0.96	0.53	11.6
		Average	22.6 ± 0.3	0.92 ± 0.03	0.48 ± 0.03	9.9 ± 0.9

OA‐PFSI also led to PV performance enhancement as *V*_oc_ increased. Nevertheless, the low FF values remained, and the spontaneous passivation effects were less than those of other OA‐based dopants (i.e., OA‐FSI and OA‐TFSI); OA‐PFSI increased *V*_oc_ to 1.00 ± 0.02 V in backward scan, from that of Li‐PFSI of 0.89 ± 0.06 V, leading to a PCE enhancement from 9.5 ± 2.1% to 12.1 ± 1.0% (Figure 6 and Table 1). In addition, OA‐PFSI increased QSS‐PCE from 3.4% to 11.2% (Figure 7c,f). However, the resulting *V*_oc_ values of the PSCs comprising OA‐PFSI (1.00 ± 0.02 V) were much lower than those of PSCs comprising OA‐TFSI (1.12 ± 0.01 V), even with a smaller energy gap between the perovskite and the HTM (Figures 4 and 5). This small increase in *V*_oc_ is attributable to the anionic nature of PFSI; electrons in PFSI are spatially more expanded than those in FSI and TFSI and frequently interact with OA cations, hampering the spontaneous perovskite passivation functions; this occurrence is in line with the results obtained for PL lifetimes (Figure 3). This trend strongly suggests that not only cationic characteristics of OA catins, the anionic nature of the counter anion in spontaneous perovskite passivators (i.e., RA‐based bis(fluorosulfonyl)imides) is also crucial for effective spontaneous perovskite passivation.

Additionally, the series resistance (*R*_s_) of PSCs provides further insights. Although direct measurement of hole mobilities of HTM layers was difficult because spiro‐OMeTAD solutions containing OA‐based bis(fluorosulfonyl)imides could not be deposited on substrates other than the perovskite layer [25], the *R*_s_ values suggest hole mobility trends of HTM layers. Figure S7 depicts *R*_s_ of the PSCs with Li‐ and OA‐based bis(fluorosulfonyl)imides HTM additives. Among the Li‐based bis(fluorosulfonyl)imides, PSCs with Li‐FSI and Li‐TFSI exhibited similar *R*_s_ values of 29.5 ± 2.4 Ω and 35.0 ± 8.6 Ω, respectively, while those with Li‐PFSI showed a substantially larger *R*_s_ value of 46.6 ± 6.9 Ω, presumably due to the non‐uniform HTM layers. On the other hand, PSCs with OA‐based bis(fluorosulfonyl)imides exhibited trends similar to those with Li‐based bis(fluorosulfonyl)imides, yet OA‐based ones resulted in lower *R*_s_ values compared to the corresponding Li‐based additives; PSCs with OA‐FSI and OA‐TFSI yielded similar *R*_s_ values of 16.7 ± 4.7 Ω and 19.8 ± 1.1 Ω, respectively, while OA‐PFSI samples exhibited a considerably larger *R*_s_ value of 31.6 ± 4.7 Ω, supporting that the non‐uniform HTM layers increased *R*_s_ values. Overall, OA‐based bis(fluorosulfonyl)imides decreased *R*_s_ relative to the corresponding Li‐based ones, despite the smaller driving energies for hole transfer from the perovskite to the spiro‐OMeTAD HTMs (Figure 5), suggesting that the use of OA‐based bis(fluorosulfonyl)imides enhanced hole mobilities of spiro‐OMeTAD HTM layers, compared to the Li‐based ones. This is presumably because the absence of counter cations in the HTM bulks facilitated the formation and stabilization of spiro‐OMeTAD cationic radicals. Moreover, the cation deficiency eliminates the interstitial space occupied by the conventional dopant cations (i.e., Li^+^), which may also contribute to the increased hole mobilities of the HTM bulk using OA‐based additives.

Overall, the effects of the Li‐ and OA‐based dopants on the PV performance indicate that energy‐alignment modulation by the anion component is a critical factor. Although deeper IEs are advantageous for obtaining higher *V*_oc_ values, which have been frequently discussed, the shallower IEs allowed by FSI‐based additives can also be beneficial for achieving higher FF values. Shallow IEs with FSI‐based additives also exhibit advantages in long‐term stability tests against humidity, which will be discussed in the next section.

**FIGURE 8:**
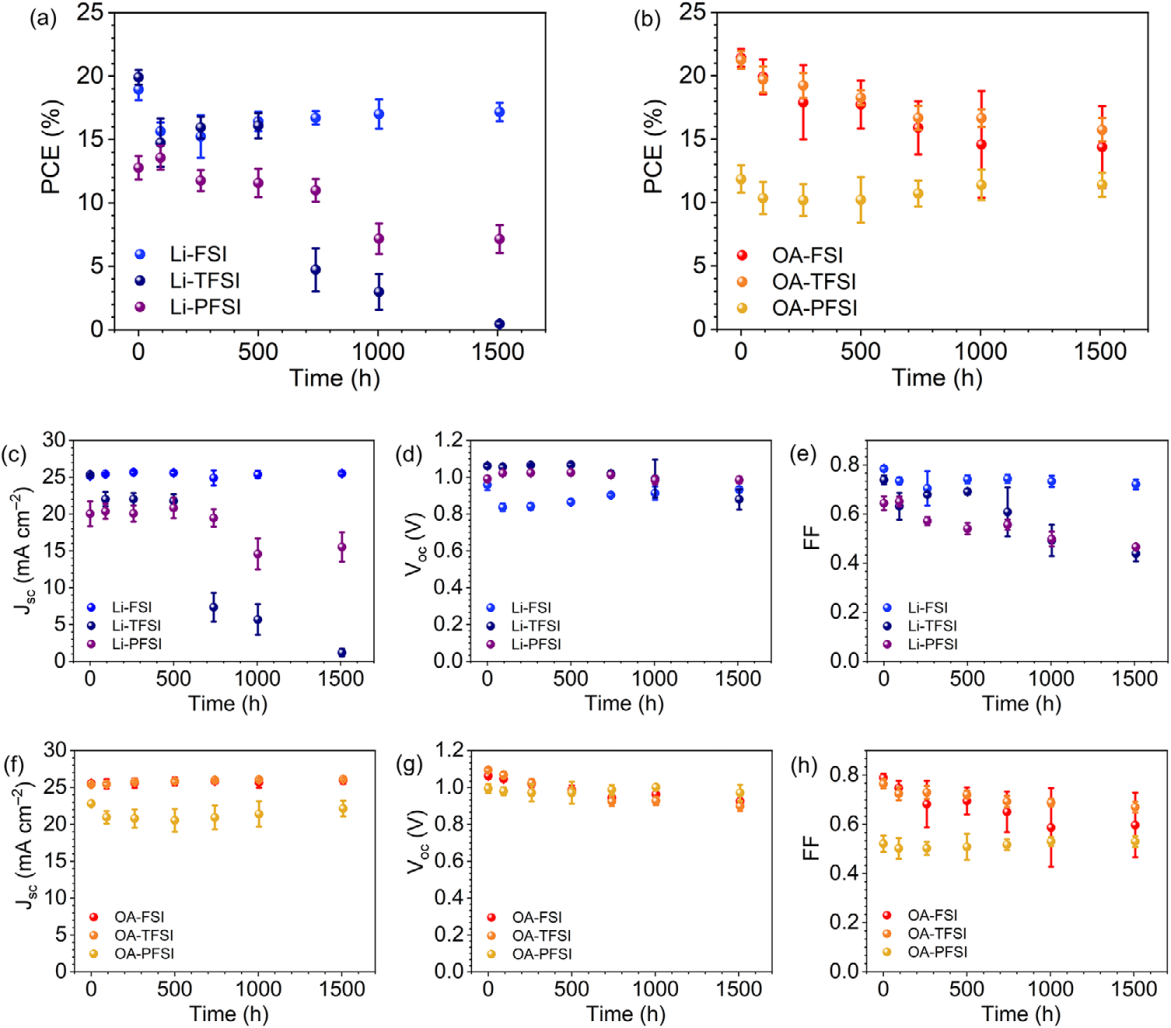
Results of long‐term stability test in the presence of humidity (50% relative humidity at 303 K (30°C)) without encapsulations. (a,b): PCE, (c,f): *J*_sc_, (d,g): *V*_oc_, (e,h): FF (Figure S7: 2nd run for the PSCs with Li‐based additives).

Furthermore, the contributions of energy alignment modulation to long‐term stability in the presence of humidity is discussed. Figure 8 illustrates the long‐term stability of PSCs at 50% relative humidity at 303 K (30°C). With Li‐series dopants, the PCEs of PSCs comprising Li‐TFSI and Li‐PFSI exhibited degradation over the duration, whereas PSCs comprising Li‐FSI very nearly maintained their PCEs over 1500 h (Figure 8a). The significant decreases in the *J*_sc_ and FF values of PSCs comprising Li‐TFSI and Li‐PFSI resulted in the observed PCE decreases. Moreover, for PSCs comprising OA‐based dopants, all PSCs mostly retained their initial PCEs over 1500 h (Figure 8b).

The high stability of PSCs comprising OA‐based dopants is in line with that reported previously [8, 23, 25, 26] Spontaneous perovskite passivation rendered the perovskite surfaces hydrophobic owing to the hydrophobicity of the OA moiety covering the perovskite surfaces, thereby improving the long‐term stability of the PSCs in the presence of humidity. The hydrophobic perovskite layer hampers the entrance of moisture into the perovskite, leading to retention of the original quality of the perovskite photoabsorbers. Indeed, the perovskite layers, after the removal of HTMs comprising OA‐based additives, exhibited a larger CAs of 72°–82° with water droplets than those with Li‐based additives with CAs of 46°–51° (Figure S3). The almost unchanged *J*_sc_ values of the PSCs comprising OA‐based HTM additives in the stability test (Figure 8f), in contrast to that of Li‐based additives, except for that of Li‐FSI, support this effect [71], that is, OA‐based HTM additives protected the perovskite layers from atmospheric moisture.

However, the trends for Li‐based dopants differ from those reported previously. Previous papers concluded that with an increase in the size of C‐F moieties in bis(fluorosulfonyl)imides (i.e., from FSI to TFSI and PFSI), the hydrophobicity of HTMs increased owing to the greater hydrophobicity of bis(fluorosulfonyl)imides, thereby improving the PSC stability against humidity [9, 10, 11]. Thus, PFSI is reportedly the most advantageous among the bis(fluorosulfonyl)imides used in terms of its stability against humidity [9, 10, 11]. In contrast, in our study, FSI exhibited the greatest stability against humidity. To examine the reproducibility, a second long‐term stability test was conducted under the same conditions used for the PSCs with Li‐based additives (Figure S8). Crucially, the results of the first run (Figure 8) were reproduced, and almost the same trends in PV parameter transitions were observed. To investigate the hydrophobicity of the HTM, the CA of water droplets on HTMs comprising additives was investigated (Figure S9). The HTM comprising Li‐FSI exhibited a slightly higher CA than those comprising Li‐TFSI and Li‐PFSI. This is perhaps because the Li‐TFSI and Li‐PFSI additives resulted in substantially deeper IEs than that caused by the Li‐FSI additive, suggesting a higher concentration of spiro‐OMeTAD cationic radicals, which are polar, rendering the HTM surfaces hydrophilic and thereby reducing the CAs of the water droplets [23]. Thus, practical HTMs comprising Li‐FSI can block moisture from entering the perovskite layers more efficiently than those comprising Li‐TFSI and Li‐PFSI. This moisture‐blocking effect is supported by the almost unchanged *J*_sc_ values of the Li‐FSI‐containing samples (Figure 8c) [71]. Although the prevailing opinion in the PSC research field is that a higher concentration of spiro‐OMeTAD cationic radicals benefits PV performance because of the reduced energy losses in hole transfer from perovskites to HTMs, this work strongly suggests that high concentrations of spiro‐OMeTAD cationic radicals can be disadvantageous in terms of the hydrophobicity of HTM layers, blocking the entry of moisture into the perovskite layers.

**FIGURE 9:**
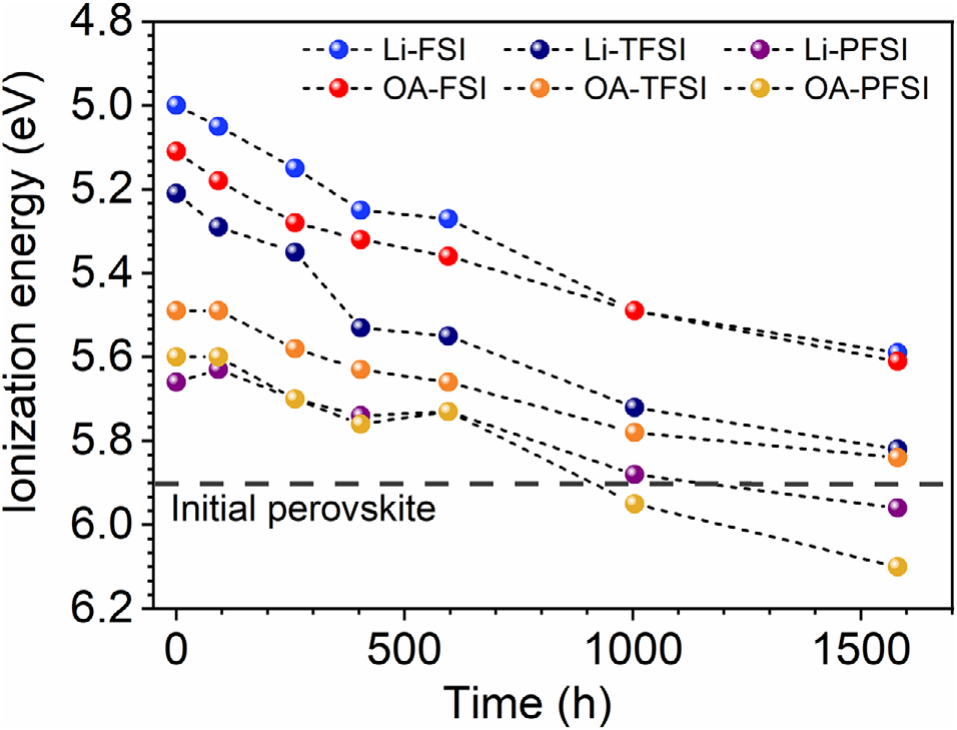
Transition of IEs of HTMs with Li‐ and OA‐based additives.

Retention of the FF values of the PSCs comprising Li‐FSI also contributed to their stable PCE, suggesting another contributing factor. The fact that moisture influences spiro‐OMeTAD oxidation is well known; hence, the time course of the IEs of the HTM layers with the dopants used in this study is of interest. Figure 9 shows the time course of the IE of the HTM layers in the long‐term stability test under the same conditions as those used for the PSCs (Figures S10 and S11: the corresponding PYS profiles). For all the samples, a longer exposure time to moisture led to deeper IEs, strongly suggesting that humidity facilitated the oxidation of spiro‐OMeTAD. Notably, the absolute IE values of the HTMs at the HTM/perovskite interface might differ from the IE values shown in Figure 9. This is because the samples were evacuated for each IE measurement, and the observed oxidation trend (Figure 9) could be slower than that for the actual PSCs. In addition, the measured IE values were for the HTM surface, where spiro‐OMeTAD oxidation was most frequently facilitated, but not for the HTM bulk and HTM/perovskite interfaces. However, the obtained IE values are indicative of the IE change trends.

Although HTMs comprising OA‐based additives initially exhibited deeper IEs than those with the corresponding Li‐based additives, the IE values which were determined by the anion component became similar after the stability test. The IEs of the HTMs comprising PFSI additives (i.e., Li‐PFSI and OA‐PFSI) were substantially deeper (∼6.1 eV) than that of the initial perovskite (5.9 eV) after the stability test, whereas those of the HTMs comprising TFSI additives (∼5.8 eV) were similar to that of the initial perovskite (5.9 eV). The actual driving energy of hole transfer from perovskites to HTMs is determined by the quasi‐Fermi level at the HTM/perovskite interface under one sun irradiation [72, 73]. When perovskite layers are degraded via exposure to moisture, the quasi‐Fermi level will be substantially shallower than the IE of the perovskite, therefore, greater driving energy will be required for hole transfer. In other words, with degraded perovskite layers, a much larger energy gap between the perovskites and HTMs is required for efficient hole transfer from the perovskites to the HTMs compared with that required for pristine PSCs. Thus, for PSCs comprising Li‐based dopants (i.e., Li‐TFSI and Li‐PFSI), the combination of reduced driving energy for hole transfer, owing to the resulting deep IEs of HTMs, and degraded perovskite layers presumably hampered hole transfer, contributing to the degradation of the PCE values, via FF decrease [71], of PSCs comprising Li‐TFSI and Li‐PFSI. However, the IEs of HTMs comprising FSI‐based additives (i.e., Li‐FSI and OA‐FSI) retained shallow IEs of ≈5.5 eV, providing sufficient driving energies for the hole transfer. The resulting shallow IEs of HTMs comprising FSI‐based additives presumably led to the higher stability of PSCs with Li‐FSI, in contrast to those with Li‐TFSI and Li‐PFSI. This observed trend highlights the benefits of shallow IEs stemming from FSI‐based additives.

## 3 Conclusion

The effects of Li‐ and OA‐based bis(fluorosulfonyl)imides, including the newly synthesized OA‐FSI and OA‐PFSI, were investigated. All OA‐based HTM additives exhibited higher PV performances than those of the corresponding Li‐based HTM additives owing to spontaneous perovskite passivation by OA cations. However, the bis(fluorosulfonyl)imide anion components determine the trends in PV performance; bis(fluorosulfonyl)imide plays a pivotal role in modulating the energy alignment in PSCs. Among FSI, TFSI, and PFSI, the larger C‐F moieties in bis(fluorosulfonyl)imides tend to cause deeper IEs, presumably owing to the spatially more expansion of delocalized electrons in bis(fluorosulfonyl)imides.

Specifically, in terms of PV performance, the FSI‐ and TFSI‐based HTM additives exhibited similar PCE, whereas their PV parameter trends were different. PSCs with TFSI‐based additives displayed higher *V*_oc_ values, most likely owing to the lower energy gap between the HTM and perovskite. Moreover, PSCs comprising FSI‐based additives exhibited higher FF values, presumably owing to the larger driving energy for hole transfer from the perovskite to the HTM, exploiting the shallow IEs of the HTM comprising FSI anions. OA‐based FSI and TFSI additives exhibited higher PCEs than Li‐based additives owing to efficient spontaneous perovskite passivation and achieved PCE of up to 22.5% and 22.6% for OA‐FSI and OA‐TFSI, respectively. The PV performance of the PFSI‐based additives was considerably lower than that of the other additives because the PFSI‐based additives degraded the morphologies of the resultant HTMs. In addition, spontaneous perovskite passivation using OA‐PFSI was less effective than that using OA‐FSI and OA‐TFSI, presumably because the spatial expansion of the delocalized electrons in the PFSI anion could frequently interact with OA cations, thereby hampering spontaneous perovskite passivation by the OA cations.

Furthermore, the effects of energy‐alignment modulation on the long‐term stability of PSCs in the presence of humidity were discussed. For PSCs with TFSI‐ and PFSI‐based additives, the IEs of the HTMs became deep, close to, or deeper than the IE of the initial perovskite after the stability tests, owing to the facilitated oxidation of spiro‐OMeTAD in the presence of moisture. As a result, the driving energy for hole transfer from the perovskite to the HTM decreased considerably, contributing to the decrease in the PCEs of the PSCs with Li‐TFSI and Li‐PFSI. This is because the perovskite layers degraded by moisture require a large amount of driving energy for hole transfer. However, the HTMs comprising FSI‐based additives exhibited the following multiple benefits and improved stability, even when using Li‐based additives: (i) the resulting lower concentration of spiro‐OMeTAD cationic radicals led to greater hydrophobicity of the HTMs, blocking the entrance of moisture into the perovskite layers, and (ii) even after the stability test, HTMs comprising FSI‐based additives retained substantial driving energies for hole transfer.

Discussions regarding the benefits of shallow IEs of HTM are rare because the prevailing opinion might be that deeper IEs are advantageous for PV performance. However, this study demonstrates some advantages of the shallow IEs of HTM allowed by FSI‐based additives, highlighting the importance of anion engineering of HTM additives and energy alignment modulation in PSCs. Furthermore, the results of anion engineering for OA‐based additives provide clues for the material design of RA‐based spontaneous perovskite passivators; anions with excessive spatial expansion of electron delocalization, such as PFSI, make efficient perovskite passivation difficult. Consequently, the insights obtained in this study provide helpful guidance for HTM additives, which can be a performance bottleneck for n‐i‐p‐structured PSCs, thereby leading to further development of PSCs.

## 4 Experimental

### 4.1 Materials

All the materials were of reagent grade and used as purchased. The formamidine hydroiodide (FAI) and mesoporous titanium oxide (m‐TiO_2_; 30NR‐R) precursors were purchased from Great Cell Solar Materials. Lead iodide (PbI_2_), *n*‐octylamine hydrochloride (OACl), lithium bis(fluorosulfonyl)imide (Li‐FSI), lithium bis(trifluoromethylsulfonyl)imide (Li‐TFSI), and lithium bis(pentafluoroethylsulfonyl)imide (Li‐PFSI) were purchased from Tokyo Chemical Industry. Methylammonium chloride (MACl) was purchased from Xian Polymer. 2,2',7,7'‐tetrakis‐(N,N‐di‐4‐methoxyphenylamino)‐9,9'‐spirobifluorene (spiro‐OMeTAD) was from Nippon Fine Chemical. All other reagents were obtained from Sigma–Aldrich. FTO‐coated transparent glass (thickness: 1.6 mm, sheet resistance ≤ 10 Ω cm^−2^) was purchased from Nippon Sheet Glass.

### 4.2 Synthesis of OA‐bis(fluorosulfonyl)imides

OA‐bis(fluorosulfonyl)imides (i.e., OA‐FSI, OA‐TFSI, and OA‐PFSI) were synthesized using an ion‐exchnage method. [23] The same molar concentration and volume of OACl and Li‐bis(fluorosulfonyl)imide (i.e., Li‐FSI, Li‐TFSI, or Li‐PFSI) aqueous solutions were prepared. The aqueous solutions were mixed and stirred for over 2 h. Subsequently, chloroform was added to this solution with stirring, and the chloroform solution was collected from the bottom of the flask. The resulting chloroform solution was subsequently washed twice with distilled water. Finally, the chloroform was removed under evaporation, followed by drying under vacuum at room‐temperature overnight. The obtained OA‐FSI, OA‐TFSI, and OA‐PFSI were liquid at room‐temperature and thus were room‐temperature ionic liquids. For the OA‐TFSI in this work, the same sample batch as that used in previous work [23] was used. The newly synthesized OA‐FSI and OA‐PFSI were characterized by NMR (Figures S12–S15). The remaining Li concentrations of OA‐FSI and OA‐PFSI were estimated by induced ion coupling to be 6.7 and < 0.2 ppm, respectively. The remaining Li amounts in OA‐bis(fluorosulfonyl)imides were negligible, indicating that the synthesis via the ion‐exchange methods were successful. The viscosities of OA‐FSI, OA‐TFSI, and OA‐PFSI were 25.0, 260, [23] and 950 mPa·s at 303 K, respectively; with an increase in the number of C‐F moieties in bis(fluorosulfonyl)imides, the viscosity of OA‐bis(fluorosulfonyl)imides increased (i.e., the order of viscosity of OA‐bis(fluorosulfonyl)imides became OA‐PFSI > OA‐TFSI > OA‐FSI).

### 4.3 Solar Cell Fabrication

The PSCs were fabricated using conventional methods [31, 54, 70] with some minor modifications. An FTO glass substrate placed on a hot plate and annealed at 723 K was coated with a TiO_2_ compact layer (∼50 nm) via spray pyrolysis using 4 vol.% titanium diisopropoxide bis(acetylacetate) in an ethanol solution. Subsequently, a m‐TiO_2_ layer was deposited by spin‐coating a mixture of 2‐methoxymethanol (8424 µL), α‐terpinol (158 µL), ethanol (4450 µL) and 30NR titanium oxide paste onto the substrate at 5000 rpm for 10 s, followed by heating the substrate at 773 K for 30 min.

A cesium‐formamidine‐lead‐iodide perovskite layer (Cs_0.06_FA_0.94_PbI_3_) was then deposited on the TiO_2_/FTO substrate by spin‐coating in a dry room (temperature: 291 K, dew point: < 243 K). The perovskite precursor solution was prepared by dissolving 1.15 m FAI, 1.32 m PbI_2_, 0.07 M CsI, and 0.46 M MACl into a mixed solvent of *N,N*‐dimethylformamide and dimethyl sulfoxide (4:1 vol ratio). The substrate was spin‐coated with this solution at 6000 rpm for 30 s, with 0.5 mL of diethyl ether added dropwise on the substrate after 15 s of spinning; subsequently, the sample was heated at 423 K for 10 min. The HTM layer was deposited by spin‐coating a solution containing spiro‐OMeTAD and additives at 3000 rpm for 30 s. The HTM solution comprising OA‐bis(fluorosulfonyl)imides (i.e., OA‐FSI, OA‐TFSI, and OA‐PFSI) was prepared by dissolving 70 mM spiro‐OMeTAD in chlorobenzene (CB), 24 mM OA‐bis(fluorosulfonyl)imides [25, 29, 30], and 96 mM 4‐*tert*‐butylpridine (tBP) [25, 29, 30]. For the HTM solution comprising Li‐bis(fluorosulfonyl)imide additives (i.e., Li‐FSI, Li‐TFSI, and Li‐PFSI), 70 mM spiro‐OMeTAD, 48 mM Li‐bis(fluorosulfonyl)imides, 210 mM tBP [74] were dissolved in CB. After HTM depositions, the samples were heated at 353 K for 10 min in a dry room. Finally, an Au conductor layer (thickness: ∼200 nm) was deposited via thermal evaporation to yield a PSC with a standard n‐i‐p structure.

### 4.4 PV Performance Measurements

Current–voltage curves were obtained using a source meter (R6243, ADVANTEST) and a class A solar simulator (XIL‐05B100KP, Seric Co.) calibrated with an Si‐reference cell equipped with a KG‐5 filter to simulate AM1.5G conditions (100 mW cm^−2^). The mismatch factor of the solar simulator was less than 25%. The measurements were conducted at room‐temperature (∼298 K) in an ambient atmosphere using a sample mask with an aperture area of 0.119 cm^2^. The current–voltage scan was performed at a constant speed of 100 mV s^−1^ under simulated sunlight. Before each measurement, an anti‐reflection sheet was pasted on the glass side of the cell. No pre‐bias was applied prior to the measurements. For each condition, more than 15 samples were measured, and the best‐performing samples were selected for analysis. The average values and standard deviations of the PV properties were calculated based on these measurements. The long‐term storage stability test was conducted for more than 12 cells using an environmental test chamber (Espec Co. Ltd.) at a temperature of 30°C (303 K) and relative humidity of 50% under dark without sample encapsulation. The EQE action spectra were measured using an action spectrum measurement setup (CEP‐99W, Bunkou Keiki).

### 4.5 Characterization

Microscopic views were obtained using a digital microscope (VHX‐7000, Keyence). The wettability of the HTM layers or the perovskite layers after removing the HTM layers was assessed using a CA meter (Kyowa Interface Science Co.; DMo‐401). PL lifetimes were measured using a time‐correlated single‐photon counting system (Fluorolog‐QM, Horiba) with an excitation wavelength of 634 nm (Delta‐diode, Horiba) and detection at 795 nm, and also estimated based on single exponential fitting using the Origin software. HTM removal for CA and PL lifetime measurements was conducted by washing the HTM/perovskite bilayer samples using 1 mL CB solution 40 times (i.e., 40 mL CB in total was used for each sample) [23]. HTM IEs were measured via photoelectron yield spectroscopy (PYS, BIP‐KV100H, Bunkou Keiki) by exploration of one‐third of the PYS curves. The remaining Li amounts in the OA‐FSI and OA‐PFSI were measured using inductively coupled plasma (ICP; ICP‐OES710, Agilent). The viscosity of the ionic liquids was measured using a viscometer (TV‐25, Toki Sangyo) at 303 K.

## Conflicts of Interest

The authors declare no conflict of interest.

## Supporting information




**Supporting File 1**: smll72464‐sup‐0001‐SuppMat.pdf.

**Supporting File 2**: smll72464‐sup‐0002‐DataFile.zip.

## Data Availability

The data that support the findings of this study are available in the supplementary material of this article.
