# Supplementary material for: Anion Component Engineering of Spontaneous Perovskite Passivators for Energy Alignment Modulations in Perovskite Solar Cells
Source: Small. 2026 Jan 25;22(16):e12937. doi: 10.1002/smll.202512937 (PMC12994551; doi:10.1002/smll.202512937)
Supplement: Supplementary file 1 — Supporting File 1: smll72464‐sup‐0001‐SuppMat.pdf. [file SMLL-22-e12937-s001.pdf]

Supporting Information

Anion Component Engineering of Spontaneous Perovskite Passivators for Energy Alignment Modulations in Perovskite Solar Cells

Naoyuki Nishimura, Hiroaki Tachibana, Takurou N. Murakami*

N. Nishimura, H. Tachibana, T. N. Murakami

National Institute of Advanced Industrial Science and Technology (AIST), 1-1-1 Higashi, Tsukuba, Ibaraki 305-8565, Japan.

E-mail: naoyuki-nishimura@aist.go.jp

1. Spontaneous perovskite passivation by RA-TFSI

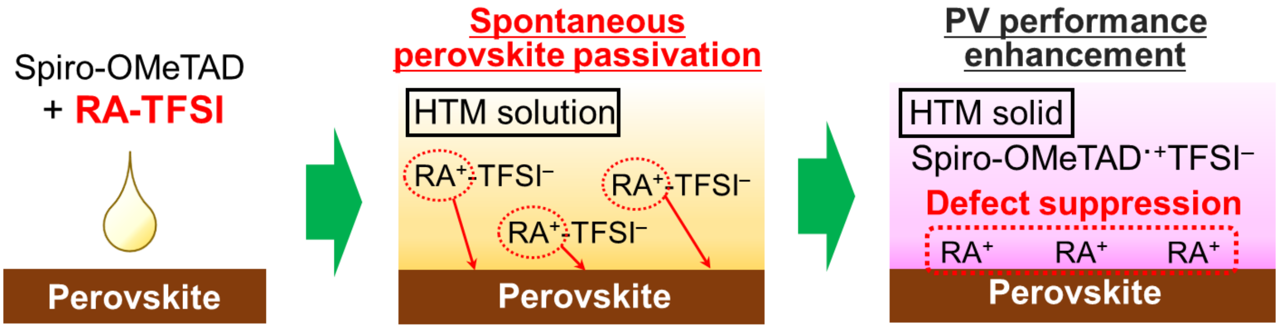

Figure S1. Schematic of spontaneous perovskite passivation during HTM deposition using an RA-TFSI-containing Spiro-OMeTAD solution; during deposition, RA cations spontaneously migrate to the perovskite surface and effectively suppress surface defects, leading to photovoltaic (PV) performance enhancement

2. Microscopic views of HTM layers with additives

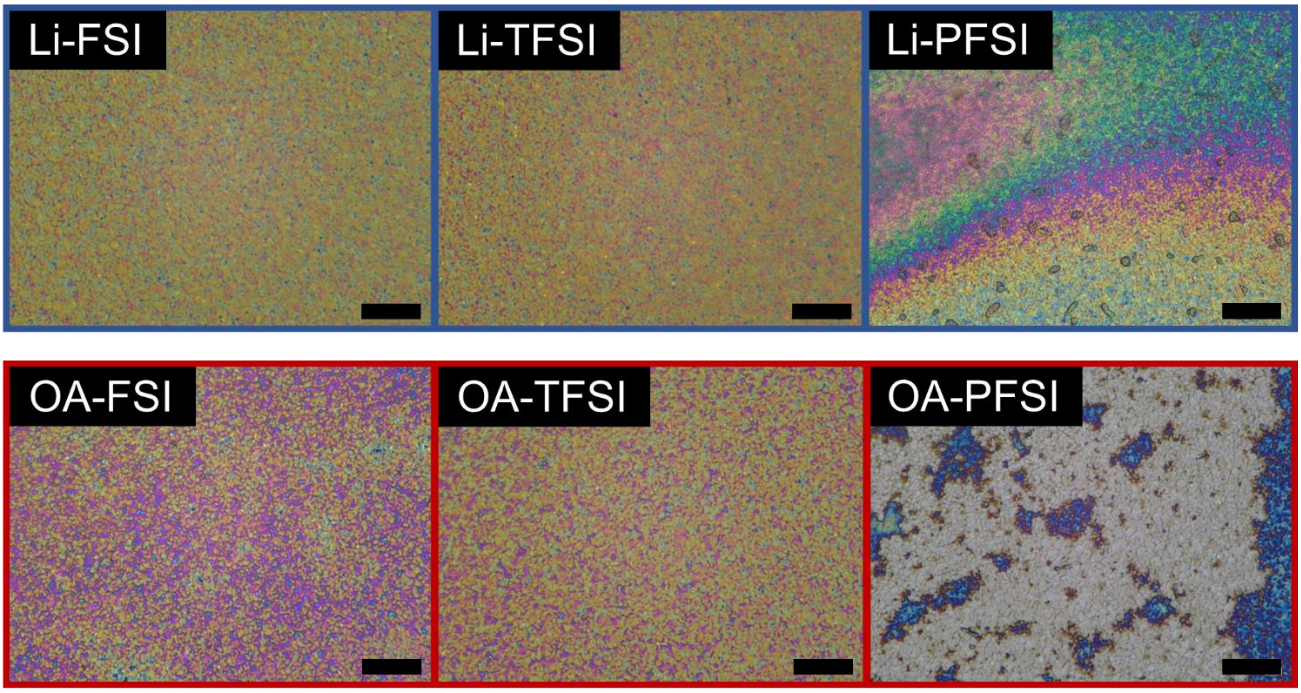

Figure S2. Magnified microscopic views of HTM layers with Li- and OA-based bis(fluorosulfonyl)imide additives; the scale bars denote 10 μm (Wider range: Figure 2)

3. Hydrophobicity of perovskite surfaces

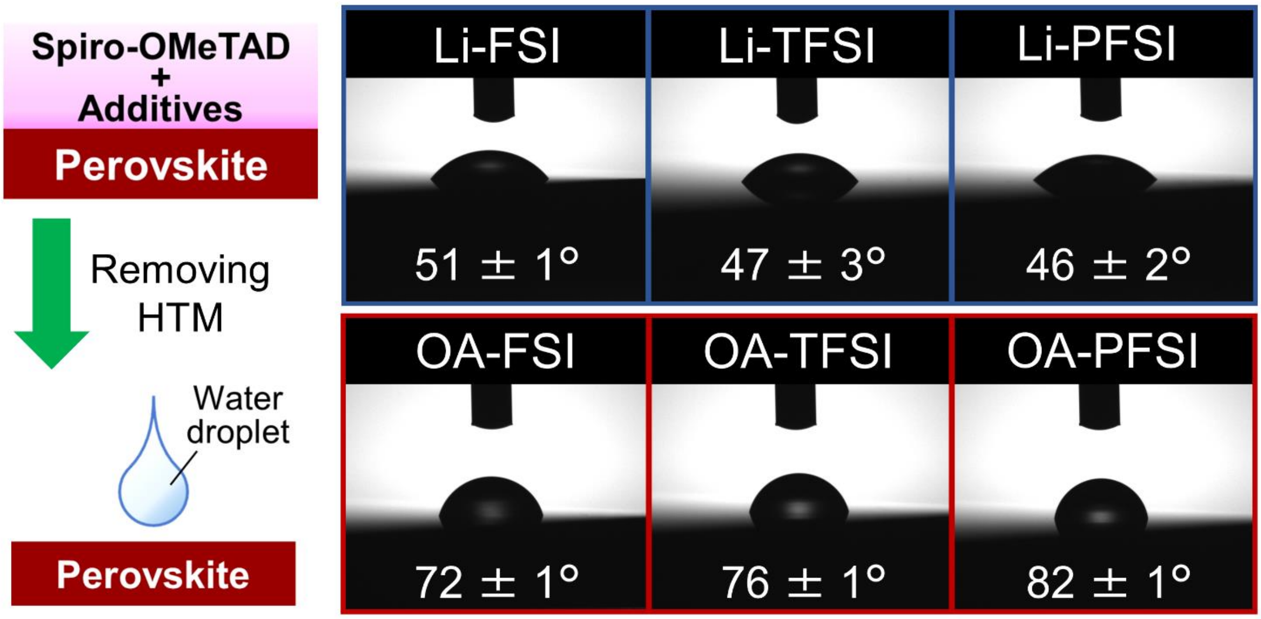

Figure S3. CAs of water droplets to perovskite layers after removal of HTMs with additives from HTM/ perovskite.

4. Ionization energy of perovskite layer

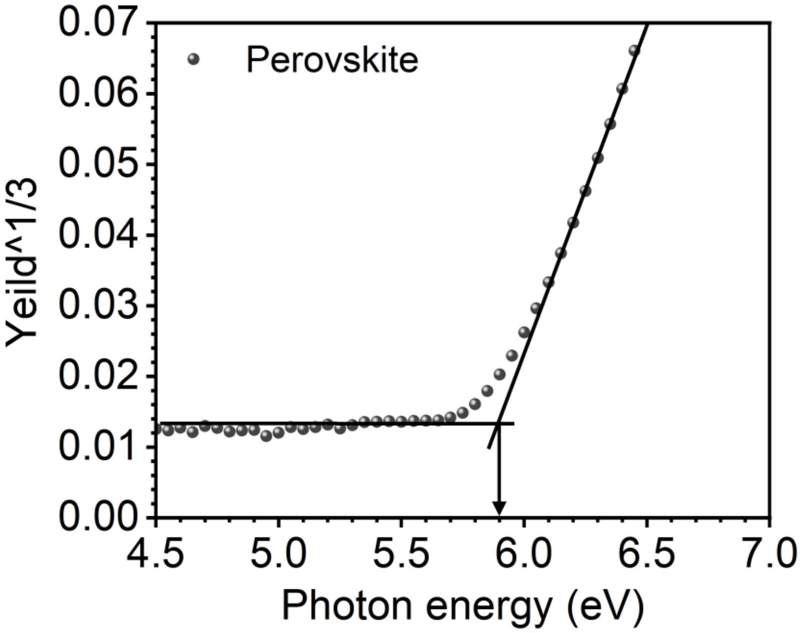

Figure S4. PYS profile of the pristine perovskite layer

5. PV performance

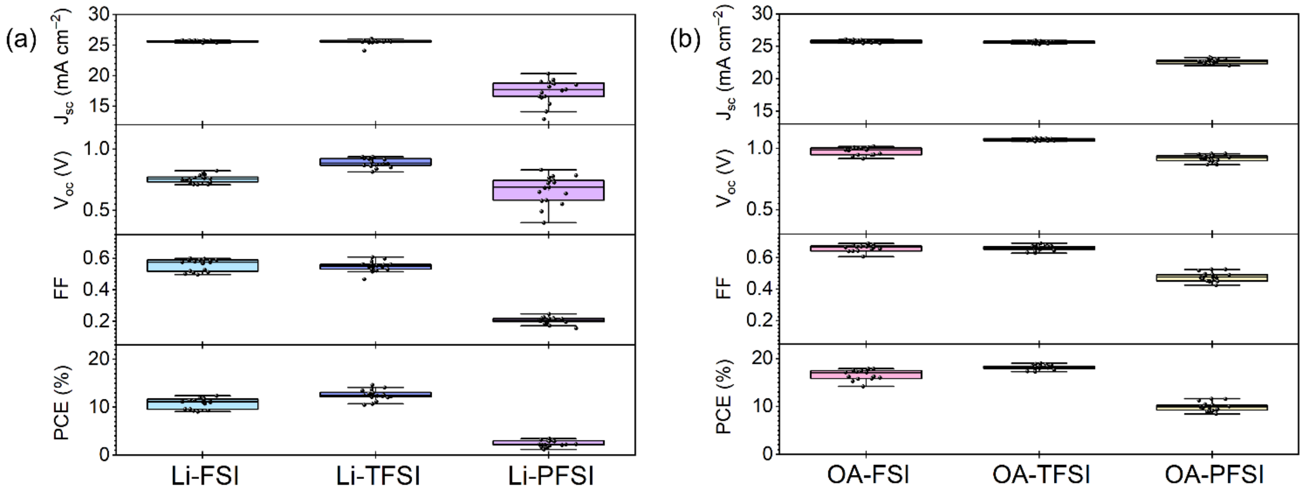

Figure S5. PV parameter distributions in forward scan for PSCs (a) with Li-based additives and (b) OA-based additives (Figure 6: backward scan).

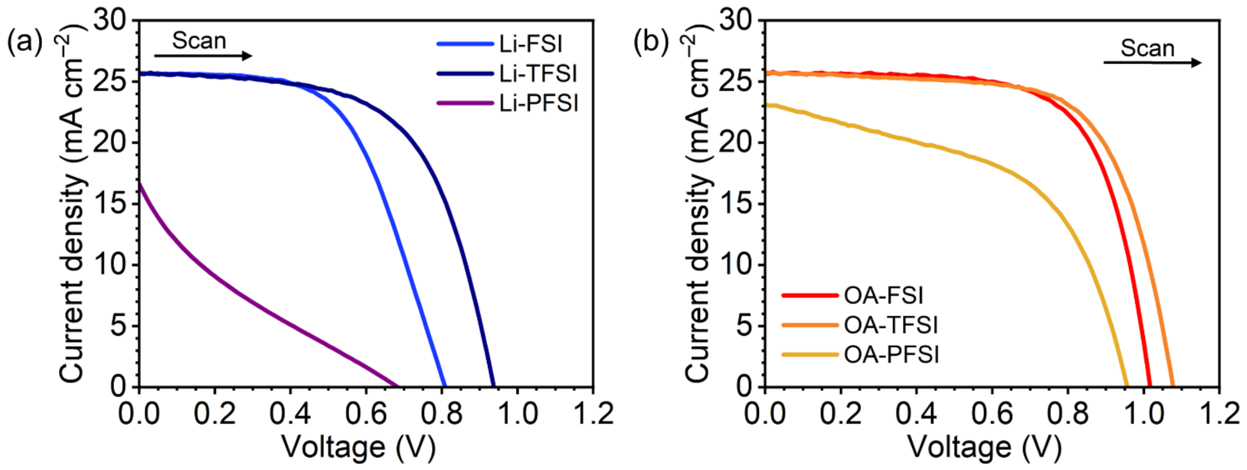

Figure S6. J-V curves in forward scan for PSCs (a) with Li-based additives and (b) OA-based additives (Figure 7a and d: backward scan)

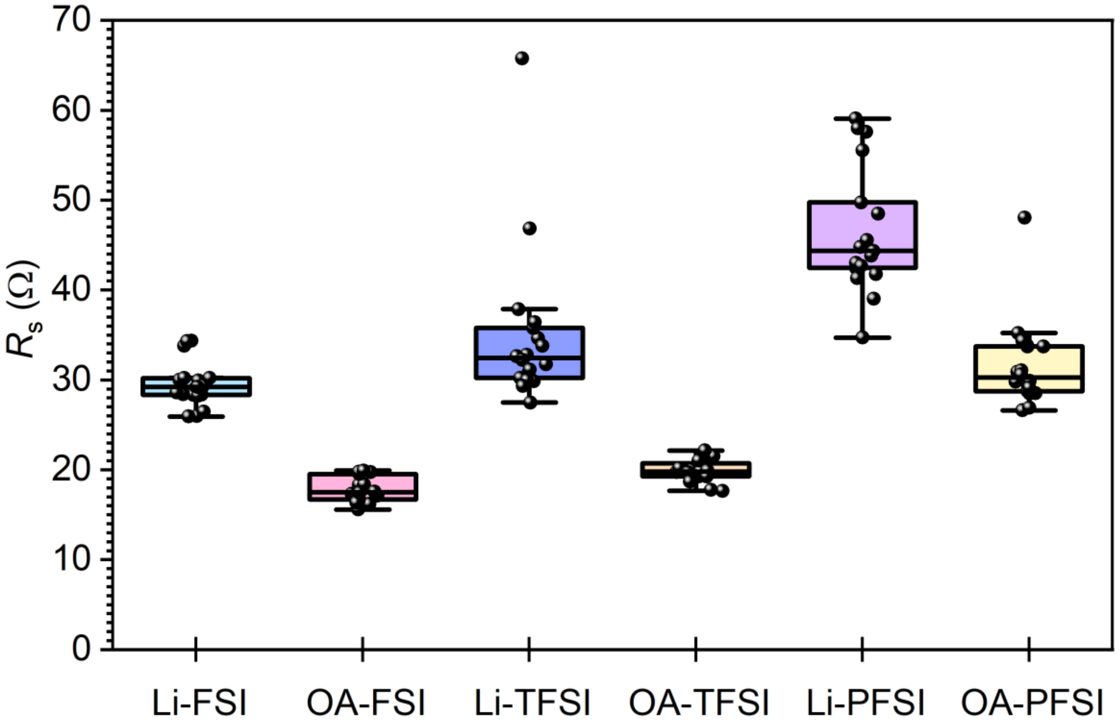

Figure S7. Series resistance (R_s) of PSCs with Li- and OA-based additives

6. Supporting data for the long-term stability test

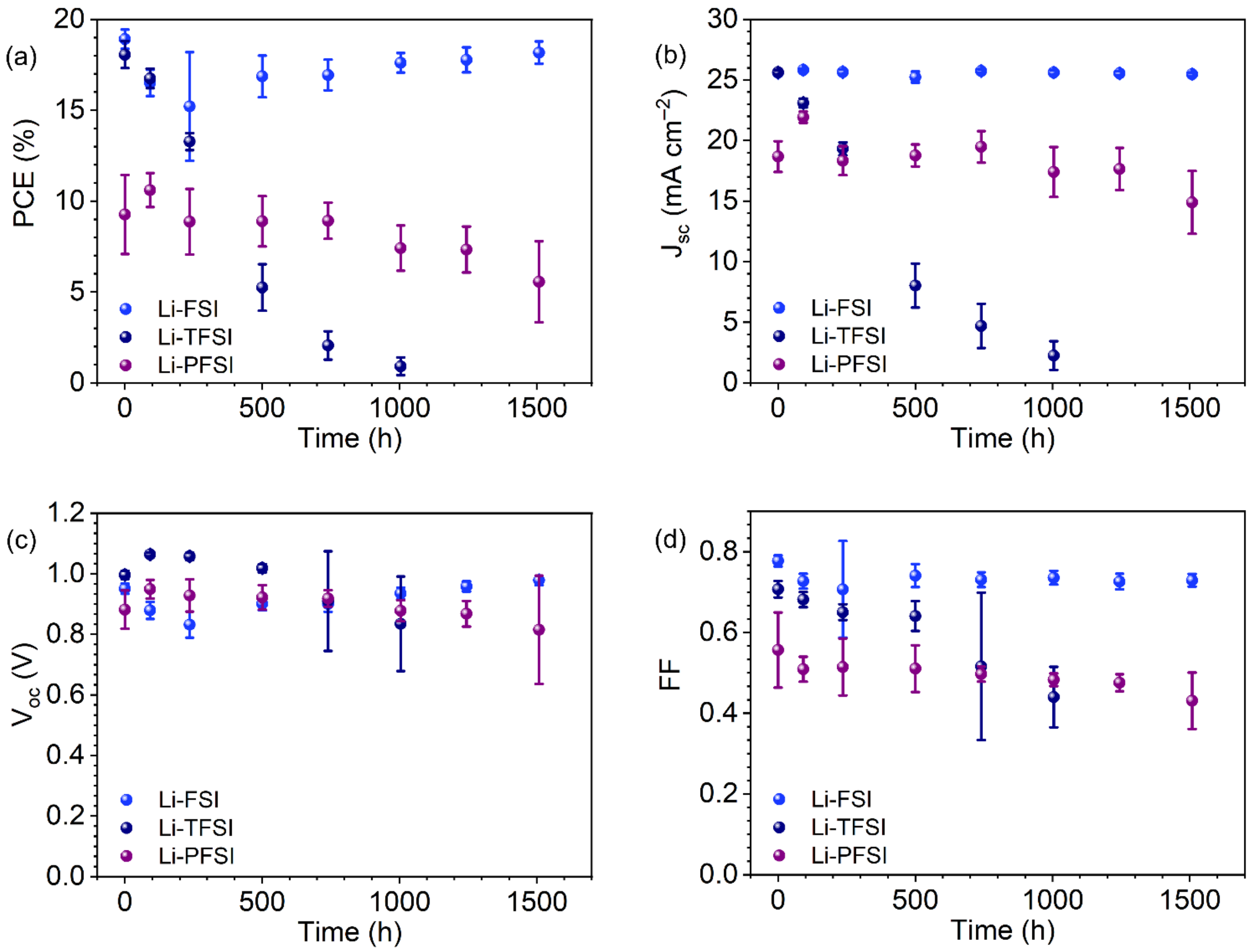

Figure S8. Results of long-term stability test (2nd run) in the presence of humidity (50% relative humidity at 303 K (30 °C)) without encapsulations. (a) PCE, (b) J_{sc}, (c) V_{oc}, and (d) FF

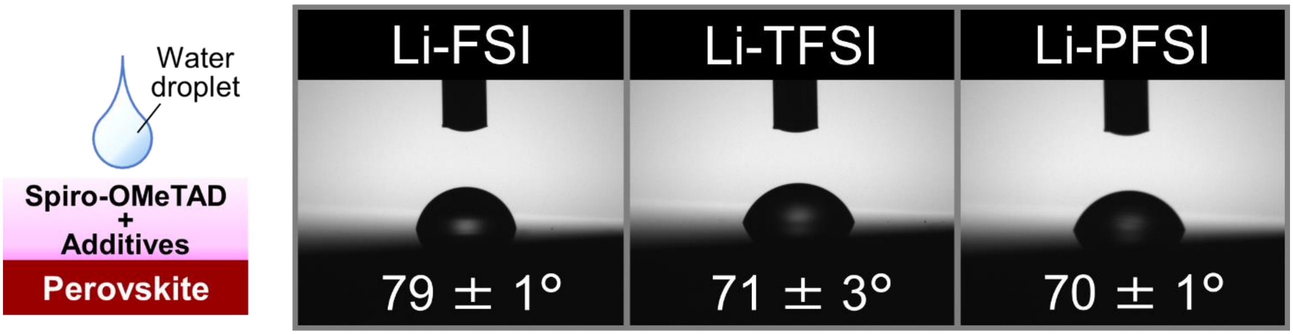

Figure S9. CA of water droplet over HTM layers with Li-based additives

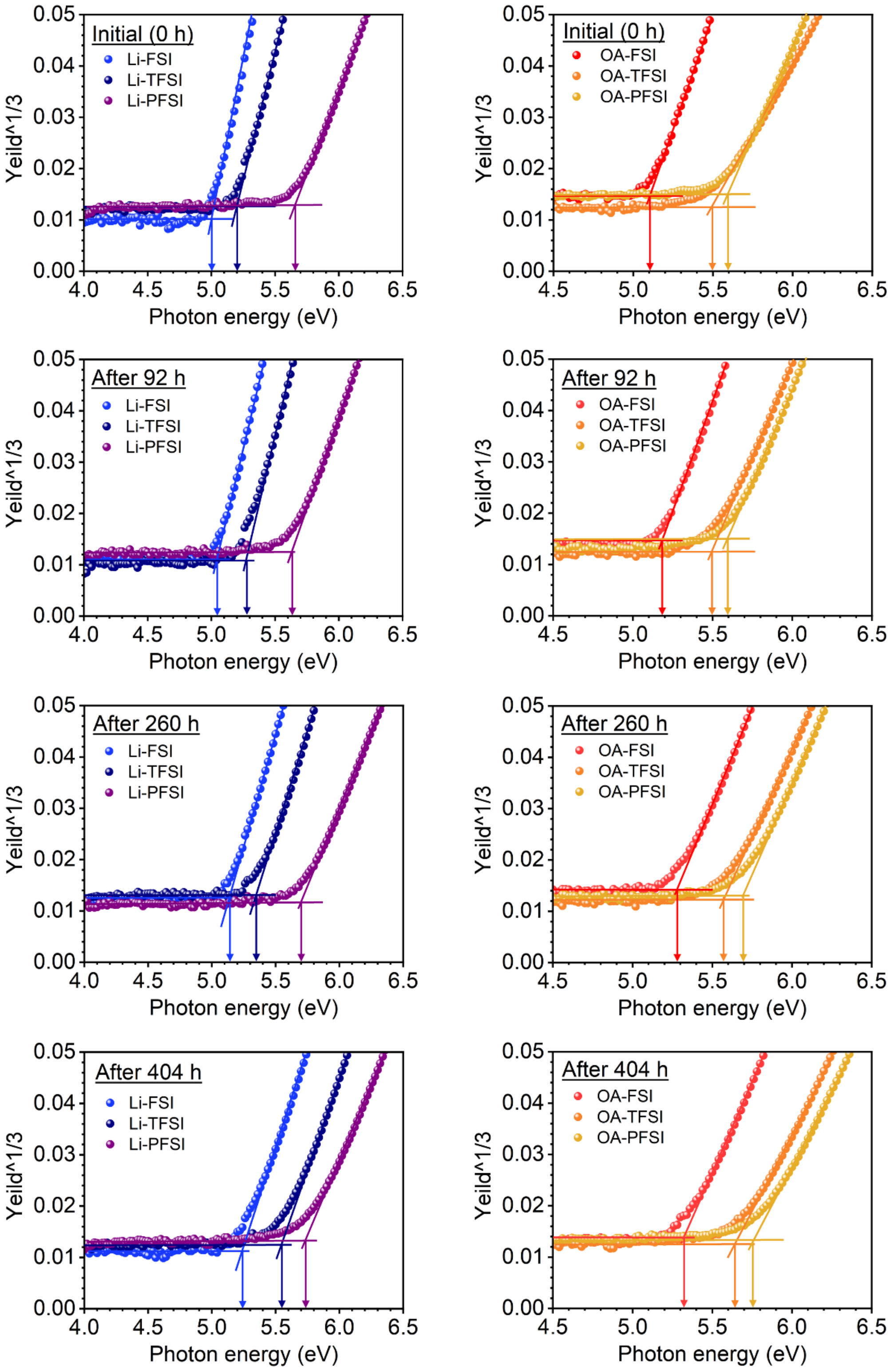

Figure S10. Transition of IEs of HTMs with Li- or OA-based additives in the stability test in the presence of humidity (50% relative humidity at 303 K) from initial (0 h) to 404 h

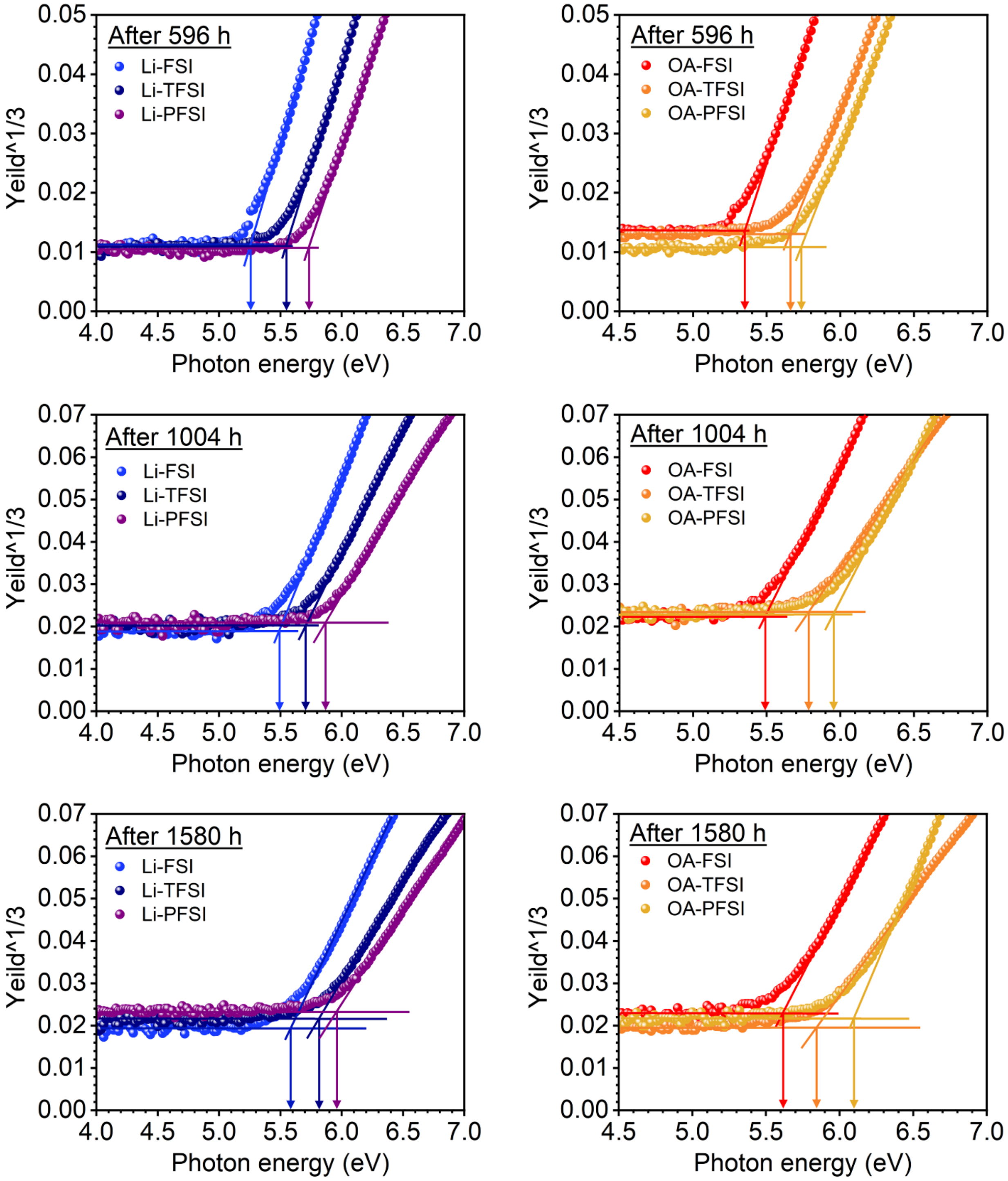

Figure S11. Transition of IEs of HTMs with Li- or OA-based additives in the stability test in the presence of humidity (50% relative humidity at 303 K) from 596 h to 1580 h

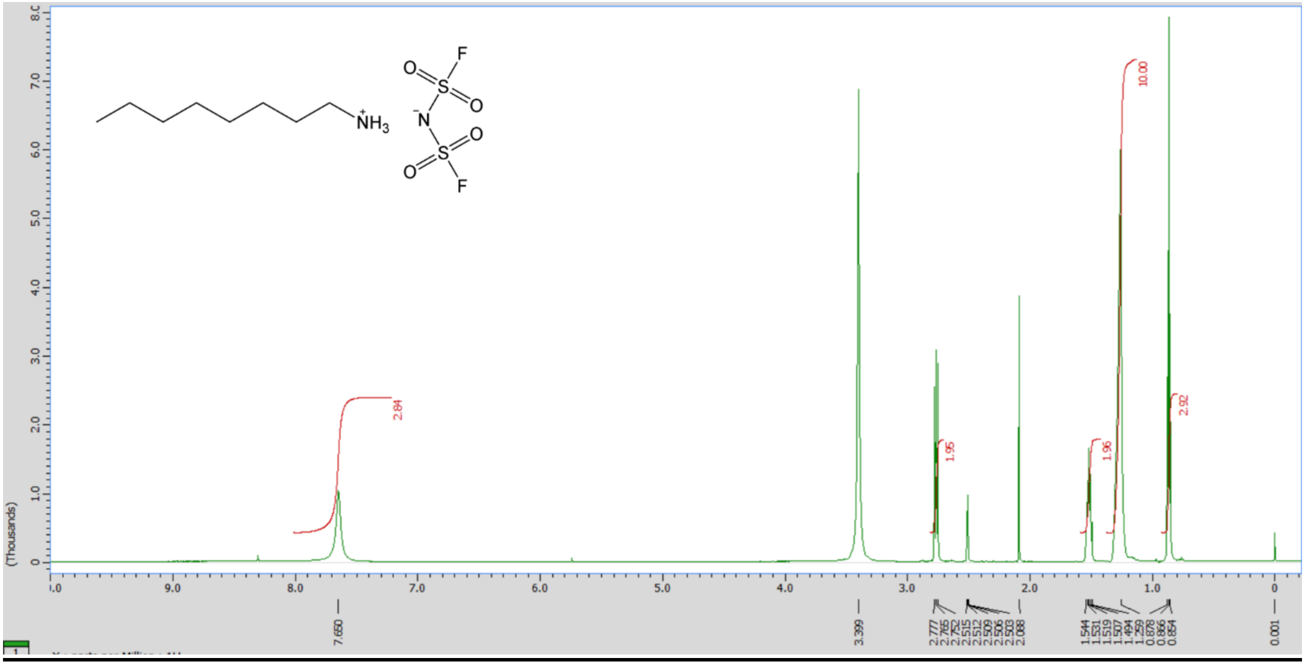

Figure S12. ^{1}H-NMR spectra of the OA-FSI; ^{1}H-NMR (400 MHz, $CDCl_3$): δ 7.65 (3H, br, CH_2**NH_3**), δ 2.75-2.78 (2H, *t*, **CH_2**NH_3), 1.49-1.54 (2H, *m*, CH_2), 1.26 (10H, m, CH_2), and 0.85-0.88 (3H, *t,* CH_3)

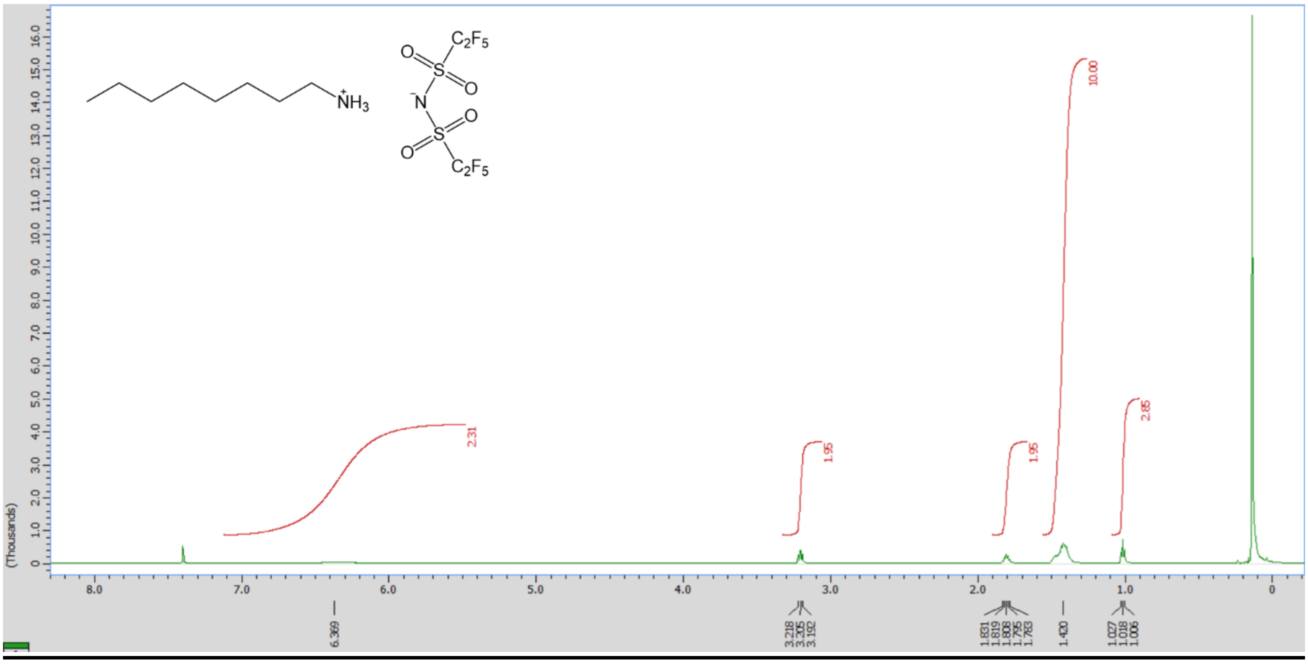

Figure S13. ^{1}H-NMR spectra of the OA-PFSI; ^{1}H-NMR (400 MHz, $CDCl_3$): δ 6.37 (3H, br, CH_2**NH_3**), δ 3.19-3.22 (2H, *t*, **CH_2**NH_3), 1.78-1.83 (2H, *m*, CH_2), 1.42 (10H, m, CH_2), and 1.01-1.27 (3H, *t,* CH_3)

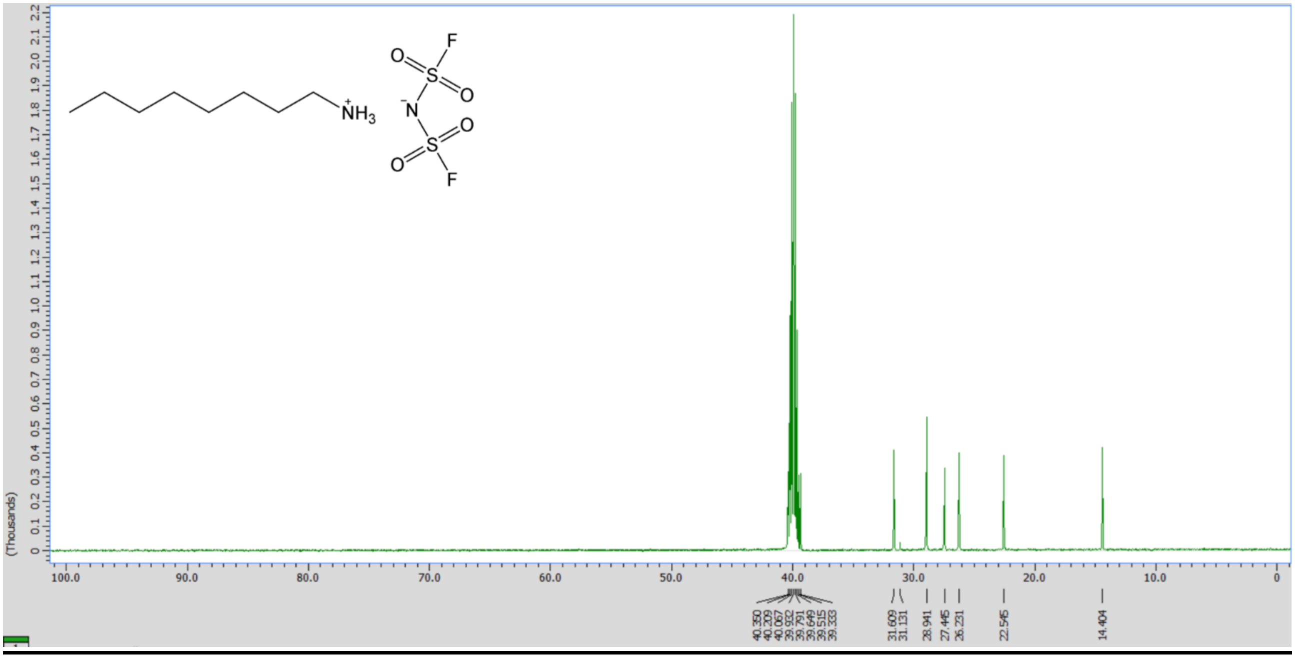

Figure S14. ^{13}C NMR spectrum of OA-FSI (100 MHz, d6-DMSO)

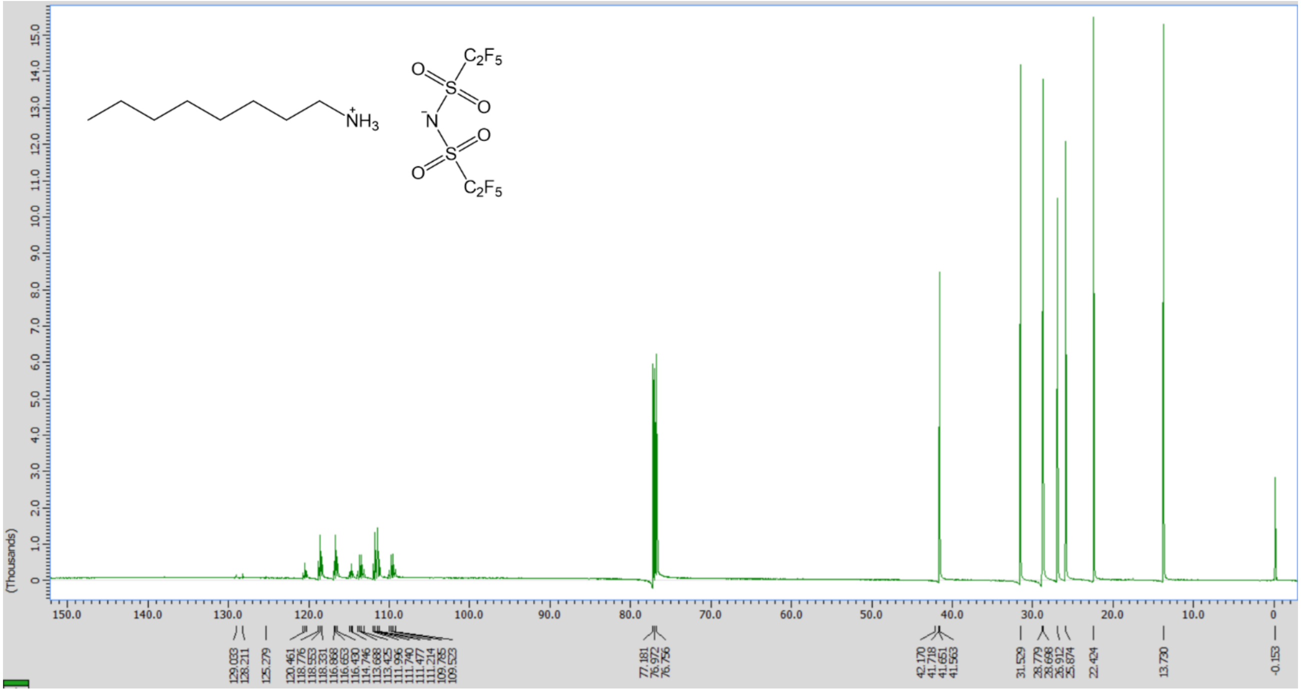

Figure S15. ^{13}C NMR spectrum of OA-PFSI (100 MHz, d6-DMSO)
